# Mimicking immune complexes for efficient antibody responses

**DOI:** 10.3389/fimmu.2025.1570487

**Published:** 2025-04-28

**Authors:** Jonathan Schönfelder, Omar El Ayoubi, Oles Havryliuk, Rüdiger Groß, Alina Seidel, Tamam Bakchoul, Jan Münch, Hassan Jumaa, Corinna S. Setz

**Affiliations:** ^1^ Institute of Immunology, Ulm University Medical Center, Ulm, Germany; ^2^ Institute of Molecular Virology, Ulm University Medical Center, Ulm, Germany; ^3^ Centre for Clinical Transfusion Medicine, University Hospital of Tübingen, Tübingen, Germany

**Keywords:** IgG, B cells, antibody responses, adjuvant, memory

## Abstract

Efficient antibody responses are crucial for combating infectious diseases and vaccination remains a cornerstone of this effort. This study introduces a novel approach for enhancing immune responses in wild-type mice by utilizing pre-formed immune complexes, using the receptor-binding domain (RBD) of SARS-CoV-2 as a model antigen to illustrate the broader potential of the concept. Specifically, we found that pre-treating the antigen with bis-maleimide, a chemical linker that facilitates protein cross-linking, significantly enhances antibody production. Moreover, *in vitro* cross-linking of antigen to unrelated IgG using bis-maleimide generated immune complexes that markedly enhanced antigen-specific antibody responses, likely by mimicking natural memory-like mechanisms, suggesting that bis-maleimide pre-treated antigens may similarly engage IgG *in vivo*. In contrast, antigen crosslinking with IgA or IgM did not yield comparable effects, highlighting the unique capacity of IgG to boost immunogenicity. By leveraging the principles of immune memory, this study demonstrates the potential of pre-formed immune complexes to significantly enhance vaccine efficacy using an antigen-independent strategy broadly applicable to diverse pathogens.

## Graphical Abstract

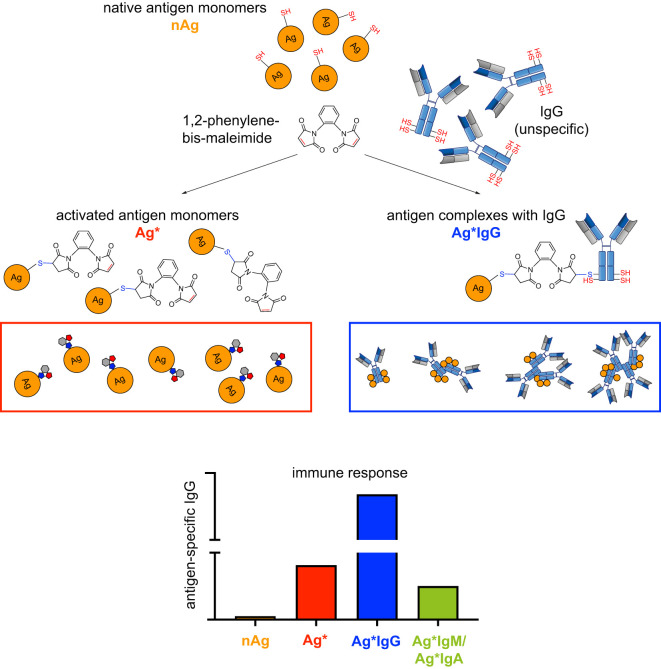

## Introduction

Protective antibody responses are crucial for the immune system’s ability to combat infectious diseases. B cells are central to this process, producing antibodies that can neutralize pathogens, thereby preventing infection and mitigating disease progression. Vaccination has been pivotal in public health, effectively reducing the incidence and severity of various infectious diseases by eliciting robust and long-lasting immune responses (1). Understanding the mechanisms that lead to efficient and protective antibody responses is essential for designing effective vaccines, particularly against emerging infectious diseases such as COVID-19.

Different antigen forms can significantly influence the quality of the immune response. Monovalent antigens, which contain a single epitope, often elicit a more focused but potentially less potent immune response compared to multivalent antigens, which present multiple copies of an epitope, thereby enhancing B cell receptor (BCR) cross-linking and activation (2, 3). Multivalent antigens have been shown to improve immunogenicity by increasing the density and variety of epitopes available to B cells, a principle utilized in several successful vaccines (4).

BCR classes, such as IgM and IgD, play distinct roles in the immune response. IgM is the first antibody isotype produced during a primary immune response and is effective at neutralizing pathogens and activating complement. IgD, although less understood, is thought to play a role in B cell activation and regulation. For example, IgD has been implicated in the initiation of immune responses and influencing the behavior of B cells during infections and immunizations. Particularly, IgD-class BCR has been shown to regulate immune responses by sensing the ratio of multivalent and monovalent antigens and to be important for efficient memory responses (5–7). During a secondary immune response, IgG, derived from IgM and IgD through class-switch recombination, predominates and provides long-term protection (8). The class-switch to IgG is crucial for enhancing the affinity and specificity of antibodies, contributing to more effective pathogen neutralization.

Immune complexes, formed when antibodies bind to antigens, are crucial in the immune response. They facilitate the clearance of pathogens by promoting phagocytosis and activating complement pathways. Moreover, immune complexes enhance antigen presentation to T cells, thereby promoting a more robust and specific adaptive immune response (9). The formation and function of immune complexes are influenced by the nature of the antigen and the class of antibodies involved, highlighting the complexity and precision required for optimal immune activation.

In our study, we chose the receptor-binding domain (RBD) in the spike protein of severe acute respiratory syndrome coronavirus 2 (SARS-CoV-2), the causative agent of the COVID-19 pandemic (10), as a model antigen to explore different immunization strategies and their efficacy in eliciting protective antibody responses. For instance, the SARS-CoV-2 spike protein binds via its RBD to angiotensin-converting enzyme 2 (ACE2) on the host cell surface, which is critical for virus entry (11). Consequently, triggering antibody responses, which block the RBD/ACE2 interaction is considered to be key for preventing coronavirus infection (12).

In this study, we tested the hypothesis that activated antigen, i.e. capable of generating immune complexes, is the most effective in inducing immune responses and that antigen cross-linking to IgG *in vitro* generates immune complexes that induce robust antigen-specific antibody responses.

## Results

### Antibody responses by native or complex RBD

**Figure 1:**
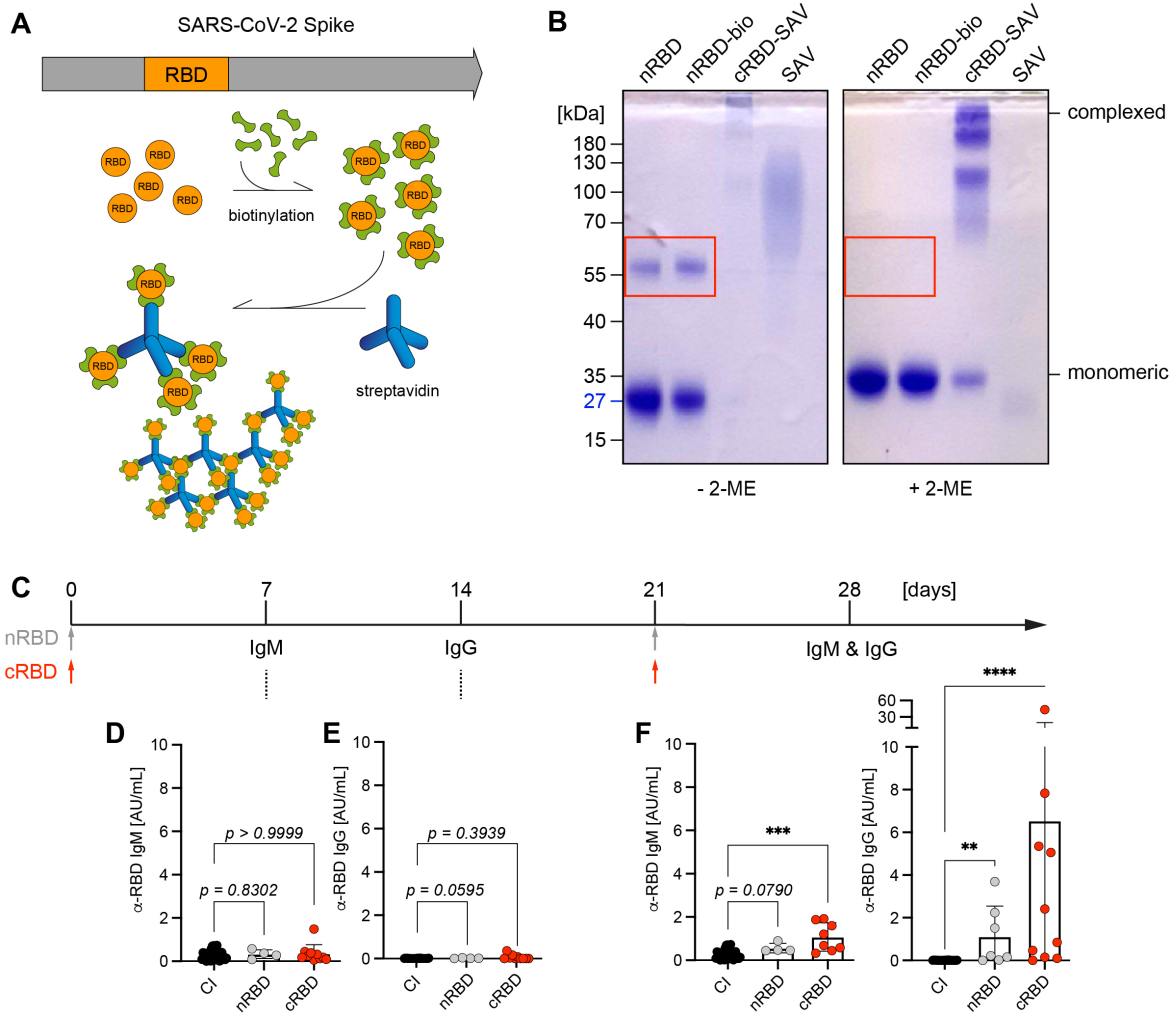
Antibody responses by native or complex RBD. **(A)** Schematic illustration of SARS-CoV-2 spike protein: Receptor-binding domain (RBD, orange), which interacts with human angiotensin converting enzyme 2 (ACE2) and thereby mediates entry of viral particles into the host cell was described as a target for neutralizing antibodies. Native RBD (nRBD) was produced in HEK293-6E cells, biotinylated and complexed with streptavidin (SAV). **(B)** nRBD (~27kDa) was biotinylated and complexed by addition of SAV, samples were separated under non-reducing (- β-mercaptoethanol (2-ME)) and reducing conditions (+ 2-ME) on a 10% SDS-PAGE and stained with Coomassie. RBD forms self-aggregates that can be dissolved by reducing disulfide bonds with 2-ME, highlighted by red rectangle. **(C)** Schematic overview of immunization procedure: WT mice were either control-immunized (CI), immunized intraperitoneally (i. p.) with 50 µg of native RBD (nRBD) or biotinylated RBD complexed with SAV (cRBD) in presence of CpG-ODN #1826 as adjuvant. Immunization was repeated on day 21 in CI, nRBD- and cRBD-immunized mice with the same vaccination composition used for primary immunization. Serum was collected and analyzed for RBD-specific antibodies on days 7, 14 and after booster vaccination on day 28. **(D–F)** Serum was harvested from immunized mice at the indicated time points after primary vaccination and RBD-specific IgM **(D)** and IgG **(E)** titers were determined by indirect ELISA. 7 days following booster immunization, serum was collected and the measurement of RBD-specific IgM and IgG levels was repeated for day 28 **(F)**. CI, n = 29; nRBD, n = 4; cRBD, n = 8 or 10, respectively. Mean ± SD, statistical significance was calculated by using the Kruskal-Wallis test.

Our published results suggested that multivalent antigen is more efficient than monovalent antigen at inducing immune responses (6, 7). To confirm this with pathogen-derived antigen, we produced recombinant RBD from SARS-CoV-2 spike protein and found that purified native RBD (nRBD) is mostly monovalent. However, under non-reducing conditions, nRBD partially forms dimers that are disrupted under reducing conditions (**Figures 1A, B**). To generate multivalent RBD, we biotinylated nRBD and incubated the resulting nRBD-bio with streptavidin to generate higher molecular complexes of RBD (cRBD) (**Figures 1A, B**). Subsequently, we compared the capability of nRBD or cRBD in inducing antibody responses after immunization. We found that injecting nRBD at day 0 and day 21 (booster immunization) induced a weak, nonetheless detectable, IgG antibody response on day 28, while the antibody response elicited by cRBD was on average 6-fold stronger (**Figures 1C–F**).

Together, these data confirm that multivalent antigen induces a stronger immune response than monovalent or bivalent antigen.

### Robust antibody responses by RBD complexes generated by chemical cross-linking

The previous experiments suggest that RBD complexes are important for eliciting immune responses. However, immunization with RBD-streptavidin (RBD-SAV) complexes also resulted in a considerable antibody response against streptavidin (**Supplementary Figure S1A**), which is not specific for the pathogen. In addition, the generation of immune complexes by biotinylating RBD and subsequent complex formation with streptavidin are unlikely to be practical for large-scale generation of vaccines. Therefore, we tested whether a method of chemical cross-linking would be capable of generating RBD complexes. To this end, we used the compound, 1,2-phenylene-bis-maleimide (thereafter referred to as 1,2-PBM), that is typically used for irreversible cross-linking via sulfhydryl (SH) groups (**Supplementary Figure S2A**). First, we tested different 1,2-PBM concentrations and incubation times to generate different ratios of complex to native RBD (**Supplementary Figures S2B-D**).

**Figure 2:**
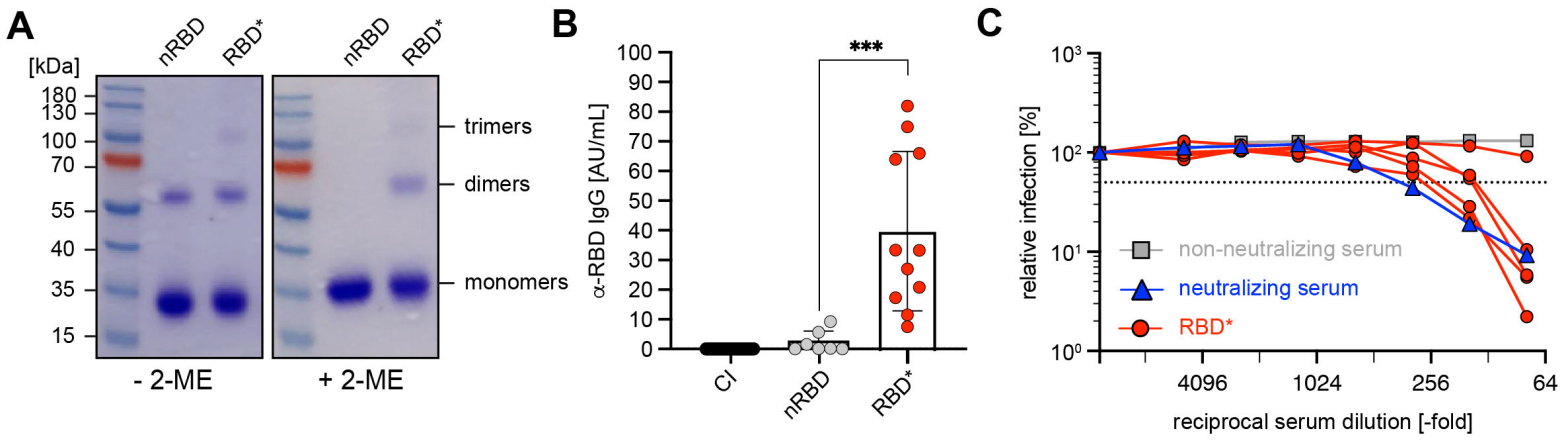
Robust antibody responses by RBD complexes generated by chemical cross-linking **(A)** Native (n)RBD (~27kDa) was produced in HEK293-6E cells and complexed by addition of 1,2-phenylen-bis-maleimide (1,2-PBM) for generation of RBD*. Samples were separated under non-reducing (- 2-ME) and reducing conditions (+ 2-ME) on a 10% SDS-gel and detected by Coomassie-staining. **(B)** Serum was collected from mice immunized with nRBD (n = 7) and RBD* (n = 11), as illustrated in **Supplementary Figure S2C** on day 28. RBD-specific IgG concentrations were measured by ELISA and compared to titers measured in CI mice. Mean ± SD, statistical significance was calculated by applying the Mann-Whitney-U test. **(C)** The neutralizing potential of generated antibodies was analyzed by a neutralization assay using pseudoviral particles in 5 serum samples from **(B)** as compared with neutralizing (blue) and non-neutralizing (gray) human serum as positive or negative control, respectively.

Cross-linking of RBD with 1,2-PBM led to stabilization of the dimers, which are spontaneously formed by nRBD. Moreover, higher molecular forms including trimers were also formed (**Figure 2A**). Next, we performed immunization experiments by injecting wild-type (WT) mice at days 0 and 21 with similar amounts of chemically cross-linked RBD (**Supplementary Figure S2C**), thereafter referred to as RBD*. The experiments show that IgM titers were hardly detectable at day 7 (**Supplementary Figure S2E**) and were moderate at day 28 (**Supplementary Figure S2F**). While anti-RBD IgG levels were hardly detectable on day 14 after primary immunization (**Supplementary Figure S2G**), secondary immunization on day 21 induced a strong increase in IgG concentration on day 28 (**Figure 2B**). These data show that chemical cross-linking produces mixtures of nRBD and cRBD that have significant capacity for the induction of antigen-specific immune responses.

Most importantly, the mice immunized with the chemically cross-linked RBD possess a high pseudovirus neutralization capacity (**Figure 2C**). These data suggest that chemical cross-linking of antigen allows simple design of efficient vaccines against pathogens including SARS-CoV-2.

### Chemically activated RBD enhances immune responses

Interestingly, the majority of chemically cross-linked RBD molecules remained as monomers (**Figures 2A**, **Supplementary Figure S2B**). We analyzed the sequence of RBD and identified a SH-group at C538, which is engaged in intramolecular disulfide bonds in full-length spike protein but not in purified RBD. We proposed that 1,2-PBM treatment of RBD may result in a substantial amount of RBD bound to 1,2-PBM in a 1:1 ratio. Thus, as a bifunctional linker, 1,2-PBM bound to RBD in 1:1 still possesses a reactive or free maleimide group, which is available for interaction and cross-linking with other proteins (**Supplementary Figure S3A**). In this configuration, RBD can be considered as an “activated antigen” ready for interaction with other components of the immune system upon injection.

**Figure 3:**
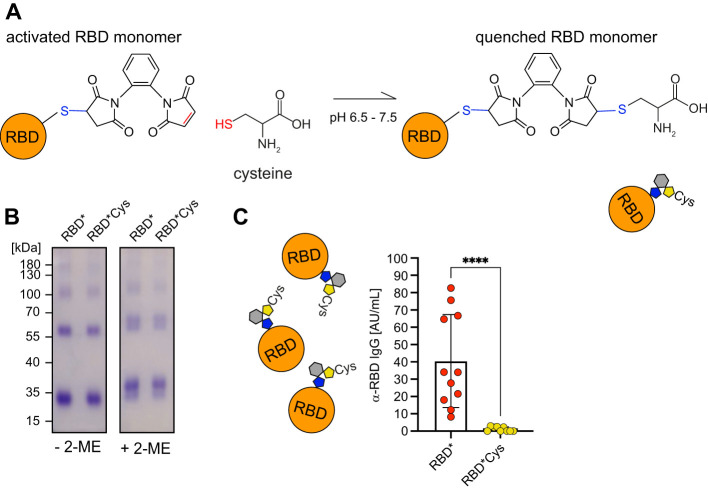
Activated RBD is required for enhanced immune responses. **(A)** Schematic illustration of the “quenching” reaction in which activated RBD* is inactivated by exposure to cysteine. **(B)** RBD was generated by addition of 1,2-PBM to nRBD. RBD* was subsequently incubated in presence of cysteine. Samples were separated under non-reducing (- 2-ME) and reducing conditions (+ 2-ME) on a 10% SDS-PAGE and detected by Coomassie-staining. **(C)** WT mice were immunized either with 50 µg of RBD* (n = 11) or “quenched” RBD*Cys, which was dialyzed before injection (n = 9). Serum was collected on day 28, one week after secondary immunization and RBD-specific IgG concentrations were measured by ELISA. Anti-RBD IgG concentrations elicited by RBD*Cys were compared to the respective concentrations, induced by RBD* immunization, which were already shown in **Figure 2B**. Mean ± SD, statistical significance was calculated by applying the Mann-Whitney-U test.

To test whether chemical cross-linking generated an activated RBD* with a free maleimide group capable of undergoing bioconjugation with other proteins *in vitro* or *in vivo*, potentially contributing to the efficient immune responses induced by RBD*, we treated the 1,2-PBM-cross-linked RBD immune complexes with cysteine *in vitro*. We hypothesized that this treatment could neutralize or “quench” the remaining reactive maleimide groups. We then assessed whether this reversion of antigen activation reduces antibody responses (**Figures 3A, B**). In fact, quenching RBD* with cysteine *in vitro* prior to immunization completely abolished the ability of RBD* to elicit anti-RBD immune responses (**Figure 3C**). Importantly, adding cysteine in acidic buffer does not change the conformation of RBD*, as RBD*Cys is recognized in a similar manner as RBD* by anti-RBD serum (**Supplementary Figure S3B**). Notably, free 1,2-PBM is not required for the immunogenicity of the activated RBD*, since antibody responses induced by RBD* were unaffected by dialysis, removing free 1,2-PBM (**Supplementary Figure S3C**).

These data show that 1,2-PBM-activated RBD* is crucial for the generation of efficient immune responses, which are most likely induced by the formation of antigen protein complexes *in vivo*.

### Pre-formed RBD-IgG complexes boost immune responses

**Figure 4:**
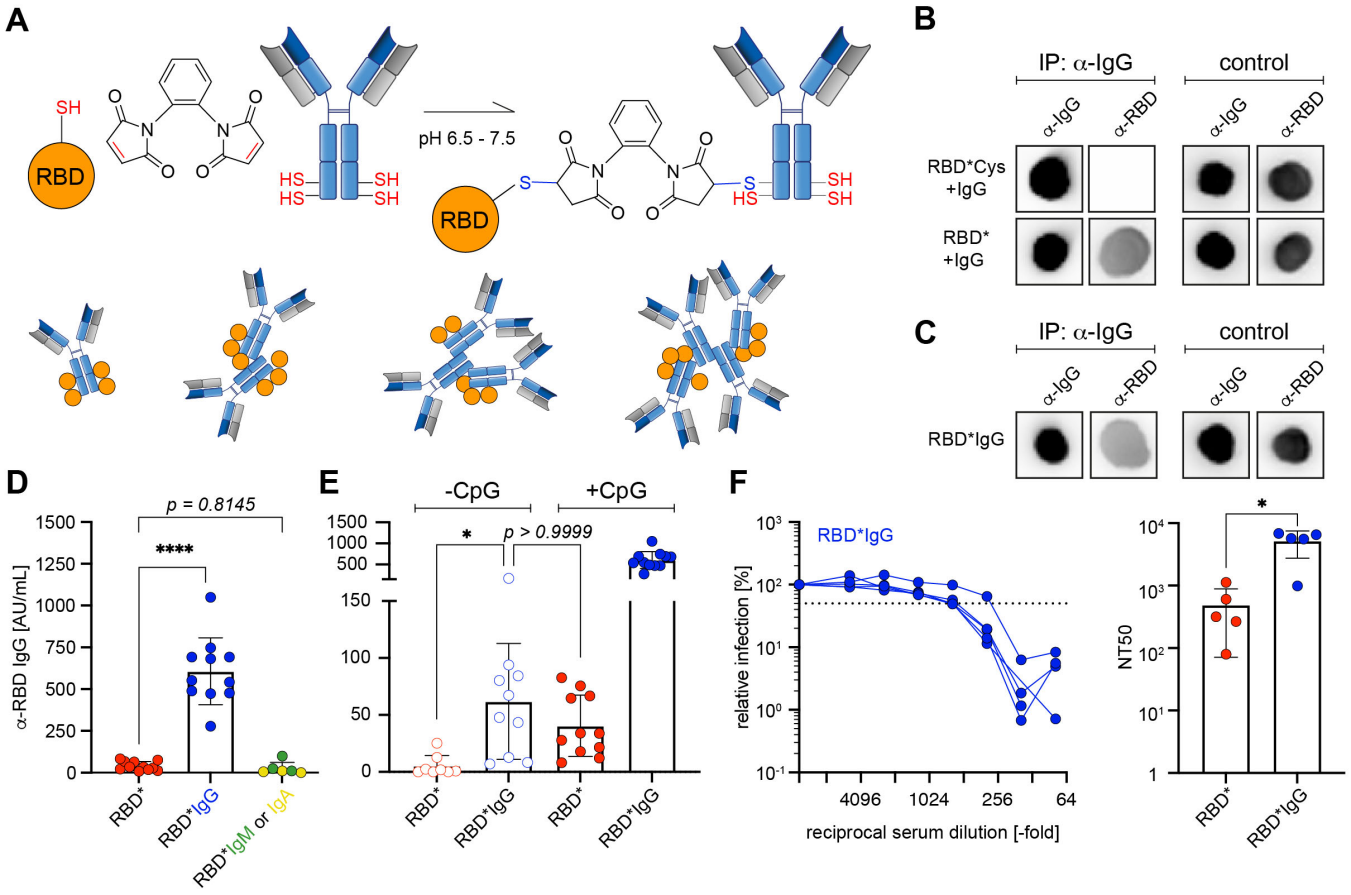
IgG is required for efficient immune responses by RBD* **(A)** Schematic illustration of cross-linking RBD* via 1,2-PBM with polyclonal murine antibodies of IgG isotype. **(B)** Dot blots of IgG immunoprecipitates against RBD. nRBD was activated with 1,2-PBM to generate RBD* and subsequently dialysed against PBS to remove free 1,2-PBM. Dialysed RBD* was either directly incubated with IgG (RBD* + IgG) or following inactivation with cysteine-solution (RBD*Cys + IgG). All samples were precipitated for IgG and developed against RBD. Representative data from 3 individual experiments are shown. **(C)** Dot blots of IgG immunoprecipitates against RBD. RBD*IgG generated by activating nRBD with 1,2-PBM in presence of IgG was used as control. Samples were precipitated for IgG and developed against RBD. Representative data from 3 individual experiments are shown. **(D)** Immunization was performed in WT mice using 50 μg of RBD* (n = 11), or RBD complexed with 1,2-PBM in presence of 25 μg polyclonal murine IgG (RBD*IgG, n = 11), IgM or IgA (RBD*IgM, n = 3 or RBD*IgA, n = 3; data already shown in **Supplementary Figure S4D**). 50 μg CpG-ODN #1826 was used as adjuvant in all conditions. Serum was collected on day 28, one week after secondary immunization and RBD-specific IgG concentrations were measured by ELISA. Anti-RBD IgG concentrations elicited by RBD* complexed with immunoglobulins of different isotypes were compared to the respective concentrations, induced by RBD* immunization, which were already shown in **Figure 2B** and **Figure 3C**. Mean ± SD, statistical significance was calculated by applying the ordinary one-way ANOVA. **(E)** Immunization was performed in WT mice as described in **Supplementary Figure S2C**, using RBD* (n = 11), or RBD*IgG (n = 11) in presence or absence of 50 μg CpG-ODN #1826 as adjuvant. Serum was collected on day 28, one week after secondary immunization and RBD-specific IgG concentrations were measured by ELISA. Anti-RBD IgG concentrations elicited by RBD* and RBD*IgG in absence of CpG-ODN #1826 were compared to the respective concentrations, induced by RBD* and RBD*IgG in presence of adjuvant, which were already shown in **Figures 2B**, **3C** and **4D**. Mean ± SD, statistical significance was calculated by applying the Kruskal-Wallis test. **(F)** The neutralizing potential of generated antibodies was analyzed by a neutralization assay using pseudoviral particles in 5 serum samples from the RBD*IgG group, shown in **(D)** and **(E)**. The 50% neutralization titer (NT50, right panel) was compared between serum samples used in the neutralization assay shown in **Figures 2C** and **4F** (left panel). Mean ± SD, statistical significance was calculated by applying the Mann-Whitney-U test.

Based on the previous results, we hypothesized that 1,2-PBM-activated RBD* undergoes bioconjugation with other proteins including immunoglobulins (Igs) (**Figure 4A**). To test this, we incubated dialyzed 1,2-PBM-activated RBD*, which was either left untreated or inactivated with cysteine solution, in presence of IgG. Subsequent immunoprecipitation for IgG and development of the eluate with anti-RBD antibodies revealed that in fact, RBD* had the capacity of undergoing covalent conjugation with IgG also upon removal of excess 1,2-PBM. Accordingly, in the condition in which RBD* was inactivated with cysteine solution prior to addition of IgG, no anti-RBD signal could be detected in the eluate (**Figures 4B, C**). To test the effect of the *in vitro* formation of antigen*IgG immune complexes, we performed 1,2-PBM-induced cross-linking of RBD in the presence of IgG. In addition, we compared the effect of IgG with that of IgM or IgA (**Supplementary Figures S4A, B**) (13). We performed immunization experiments after generating immune complexes with the different Igs (**Supplementary Figures S4C, D**). Interestingly, while IgM and IgA failed to boost the immune response, cross-linking of RBD with IgG *in vitro* led to an approximately 15-fold increase of the RBD-specific antibody response (**Figure 4D**, **Supplementary Figure S4D**). Next, we tested complex formation by other Ig isotypes when treated with 1,2-PBM in presence of RBD. Our results show that all tested Igs possess similar capacity to form complexes (**Supplementary Figure S4E**), suggesting that the enhancement in the humoral response observed upon cross-linking of RBD with Igs is a unique feature of IgG.

The enhancement observed upon using *in vitro* pre-formed RBD-IgG immune complexes prompted us to test whether the chemically cross-linked IgG induces a synergistic effect during immunization even in absence of the adjuvant CpG. To this end, we compared the immune responses of RBD* with RBD*IgG, injected in presence or absence of CpG as adjuvant. In fact, even in absence of an adjuvant, RBD*IgG induced an approximately 12-fold higher anti-RBD response as compared with RBD*, (RBD* -CpG Ø 5.37 AU/mL as compared with Ø 61.96 AU/mL in RBD*IgG -CpG; **Figure 4E**). Notably, adding CpG to the RBD*IgG mixture significantly augmented the RBD-specific antibody response (**Figure 4D, E**). Most importantly, the generated antibody responses were highly efficient in virus neutralization experiments *in vitro* (**Figure 4F**).

These results show that *in vitro* cross-linking of an antigen with IgG generates immune complexes that are highly capable of inducing robust antibody responses even in the absence of conventional adjuvants.

Complexation of RBD via biotin-SAV resulted in considerable generation of antibodies against SAV (**Supplementray Figure S1**). However, upon activation with 1,2-PBM we neither detected significant induction of antibodies directed against the bis-maleimide linker, nor against IgG used to generate the complexes (**Supplementary Figures S5A, B**). In addition, we tested whether a potential anti-IgG response might lead to elimination of antigen in recall immunizations. Therefore, we measured the anti-RBD IgG responses following a second booster vaccination on day 56 (**Supplementary Figure S5C**). The data reveal that the titers decreased after the first booster but were elevated again after the second booster, suggesting that the presence of antibodies directed against RBD (or IgG) does not affect the generation of a humoral immune response. Moreover, immunized mice did not exhibit elevated levels of systemic pro-inflammatory cytokines, as determined by measurement of IL-6 (**Supplementary Figure S5D**).

The total frequency of germinal center (GC) B cells and plasma cells (PC) was not elevated upon immunization, however, we detected significantly increased percentages of RBD-specific cells in the population of GC B cells following immunization with RBD*IgG (**Supplementary Figure S6**). Together, these data indicate that pre-formed immune complexes enhance immunogenicity without disrupting germinal center formation during the development of normal memory responses.

### IgG immune complexes act with other adjuvants

**Figure 5:**
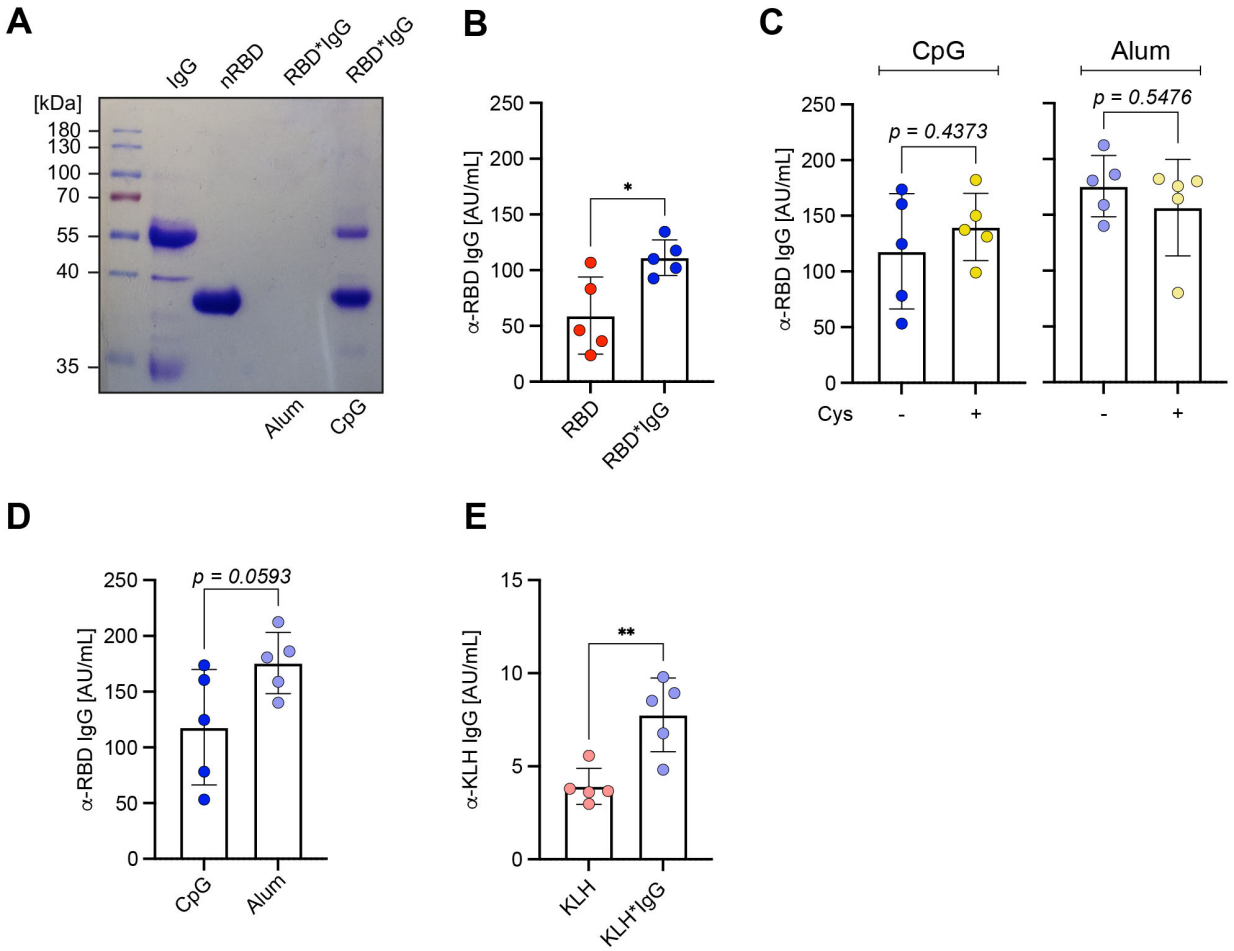
IgG immune complexes act with other adjuvants. **(A)** RBD*IgG was generated as described previously. RBD* was subsequently incubated in presence of either Alum or CpG. Samples were separated under reducing conditions (+ 2-ME) on a 10% SDS-PAGE and detected by Coomassie-staining. **(B)** RBD*IgG complexes were generated according to the previously described standard procedure. Prior to immunization, Alum was added to the previously prepared complexes and both nRBD or KLH, were used as controls. Serum was collected from immunized mice on day 28, one week after secondary immunization and RBD-specific IgG concentrations were measured by ELISA. n = 5, mean ± SD, statistical significance was calculated by applying the paired t test. **(C)** RBD*IgG complexes were generated as previously described and incubated in presence or absence cysteine (Cys). Immunization was performed in WT upon addition of either CpG or Alum as adjuvants. Serum was collected on day 28, one week after secondary immunization and RBD-specific IgG concentrations were measured by ELISA. n = 5, mean ± SD, statistical significance was calculated by applying the paired t test or Mann-Whitney-U test. **(D)** Immunization was performed in WT mice using 50 μg of RBD complexed by 1,2-PBM in presence of 25 μg polyclonal murine IgG, and subsequent addition of either CpG or Alum as adjuvant. Serum was collected on day 28, one week after secondary immunization and RBD-specific IgG concentrations were measured by ELISA. n = 5, mean ± SD, statistical significance was calculated by applying the paired t test. **(E)** KLH*IgG complexes were generated according to the described standard procedure with KLH at a concentration of 100 µg per mouse. Prior to immunization, Alum was added to the previously prepared complexes and untreated KLH was used as control. Serum was collected from immunized mice on day 28, one week after secondary immunization and RBD-specific IgG concentrations were measured by ELISA. n = 5, mean ± SD, statistical significance was calculated by applying the paired t test.

Next, we tested if the *in vitro* formation of antigen*IgG immune complexes acts independent of the type of adjuvant used. To this end, we tested Alum as a commonly used adjuvant together with the RBD*IgG immune complexes. Interestingly, we found that incubating RBD in the presence of Alum resulted in high molecular complexes that were too large to enter the gel (**Figure 5A**). We compared the immune responses after immunization with RBD using Alum in presence or absence of IgG and found that also with Alum, presence of IgG in the complex led to stronger immune responses as compared with RBD only despite the fact that Alum forms high molecular antigen complexes independent of cross-linking (**Figure 5B**). In addition, we confirmed that inactivating free maleimide groups with cysteine after formation of the immune complexes has no significant effect on the immune response against RBD (**Figure 5C**) and that Alum and CpG evoke similar immune responses when used as adjuvant together with RBD*IgG *in vitro*-formed immune complexes (**Figure 5D**). Moreover, an increase in the antigen-specific antibody response was also observed for KLH when immunized with *in vitro* formed KLH*IgG complexes suggesting that the enhancement of immune responses by *in vitro* pre-formed antigen*IgG is independent of the antigen (**Figure 5E**).

Together, these data suggest that antigen*IgG complexes generated *in vitro* provide vital approaches for the induction of strong antibody responses.

### No cytotoxicity or autoantibody production by IgG immune complexes

**Figure 6:**
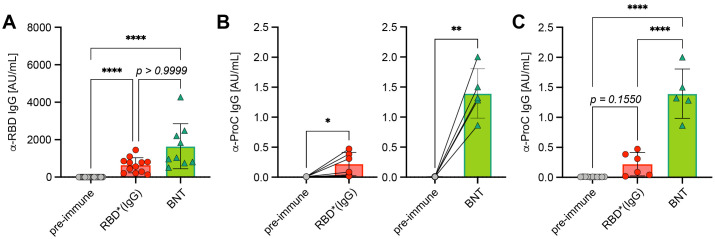
Autoreactive antibodies upon immunization. **(A)** Anti-RBD IgG concentrations were compared in serum from mice immunized twice with Comirnaty^®^ from BioNtech/Pfizer (BNT, n = 9) and RBD*/RBD*IgG (+CpG, n = 12), which reached the highest concentrations in previous experiments. Pre-immune (n = 22), mean ± SD, statistical significance was calculated by applying the Kruskal-Wallis test. **(B)** Paired comparison of anti-ProC IgG concentrations in mice prior to and after RBD*/RBD*IgG (+CpG) immunization (left, n = 6) or Comirnaty^®^ immunization (right, n = 5), respectively. Mean ± SD, statistical significance was calculated by applying the paired t test. **(C)** Grouped comparison of anti-ProC IgG concentrations from **(C)** Mean ± SD, statistical significance was calculated by applying the ordinary one-way ANOVA.

Next, we tested if the 1,2-PBM-generated antigen*IgG complexes have any effects on cell survival *in vitro* or induce risky autoimmune reactions as compared with other immunization techniques. In fact, no cell death was observed when incubating fibroblasts (HEK293) *in vitro* at concentrations of up to 10 µg/mL of 1,2-PBM-generated RBD* complexes (**Supplementary Figure S7**). However, available vaccines from different companies (BNT, MOD, AZ, JJ) induced cell death to different extent with BNT showing the highest rate of cell death despite remarkably low spike protein expression (**Supplementary Figure S8**). When comparing the immune responses *in vivo*, 1,2-PBM-generated RBD* complexes induced insignificantly weaker antibody responses as compared with BNT vaccine (Ø 634.5 AU/mL and 1651 AU/mL, respectively; **Figure 6A**). To test the effect of immunization on the generation of autoantibodies we selected protein C (ProC), a regulator of blood coagulation and inflammation (14–16), because we found increased ProC-specific autoantibodies in mice showing elevated RBD-specific titers (**Figures 6A, B**). Interestingly, we detected a significant increase in anti-ProC IgG titers in mice after vaccination with BNT as compared with RBD* or RBD*IgG (Ø 1.39 versus 0.22 AU/mL; **Figure 6C**). To confirm that high antibody titers induced by vaccination or infection are associated with increased autoreactive antibody responses, we compared anti-RBD and anti-ProC titers in a cohort of COVID-19 patients with mild or severe course of disease based on the requirement of intensive care unit treatment and found a significant increase in anti-ProC autoantibodies in patients with increased anti-RBD antibodies (Ø 0.08 in low anti-RBD IgG donors versus 0.17 AU/mL in high anti-RBD IgG donors; **Supplementary Figure S9**).

In conclusion, these data suggest that similar to infections, immunizations are associated with the risk of inducing autoreactive antibody responses which might lead to unpredicted complications. However, the use of *in vitro* pre-formed antigen*IgG seems to produce robust antibody responses while the increase in autoantibody responses is moderate as compared with other methods.

## Discussion

This study explores various immunization protocols to determine how to trigger an efficient and protective immune response, using the RBD of SARS-CoV-2 as a model antigen. The aim is to establish protocols that could be applicable to other pathogens and to compare these protocols with available procedures.

We explored the use of the entire RBD domain, both in its native form and as part of higher molecular complexes formed through biotinylation and streptavidin incubation. The immune responses induced by these complexed antigens were stronger as compared to the native RBD, but still not sufficient for virus neutralization. This indicates that an optimal immune response requires a balance between complex and monomeric antigens. Research has shown that antigen presentation can significantly impact the efficacy of the immune response, with complex antigens often providing better immunogenicity due to their ability to present multiple epitopes simultaneously (17–19).

A significant discovery of the study showed that activated antigens, which can interact with proteins *in vivo*, are the most effective in inducing immune responses. Using 1,2-phenylene-bis-maleimide (1,2-PBM) to chemically cross-link RBD produced stable complexes that maintained significant immunogenicity. These chemically cross-linked antigens induced strong IgG responses and demonstrated high neutralization capacity *in vitro*. The ability of these activated antigens to interact with proteins *in vivo* suggests that they may mimic the natural process of antigen recognition and immune complex formation, thereby enhancing the overall immune response.

We also investigated the role of different immunoglobulins (IgG, IgM, IgA) in enhancing immune responses. Only cross-linking RBD with IgG, but not with IgM or IgA, significantly boosted the antibody response. This suggests that IgG-containing immune complexes play a crucial role enhancing antigen-specific immune responses, in contrast to protein complexes even if they contained immunoglobulins of isotypes other than IgG (comparison **Figures 1F**, **4D**). It is tempting to speculate that the observed enhancement of antibody responses elicited by IgG-containing RBD complexes is induced by IgG effector functions that are activated upon recognition by Fc-gamma receptors expressed on various immune cells (20–22). Fc receptors link functions of humoral and cellular immunity. By binding antibodies that opsonize pathogens they facilitate phagocytosis and are crucial for mediating antibody-dependent cell-mediated cytotoxicity (23, 24). While Fc-gamma and -epsilon receptors have been extensively studied, the role of Fc-mu or -alpha receptors still remains unclear (25). Our results show that although complexes seem to be formed by different Ig isoforms to comparable extent, complexes comprising only IgM or IgA do not boost the antibody response as compared with IgG. This suggests that the functions of Fc-mu or -alpha receptors significantly differ from those described for Fc-gamma receptors. Interestingly, in humans and mice different types of Fc-gamma receptors exhibit differential properties in terms of binding to the individual IgG subclasses (26). Therefore, it is conceivable that in mice particularly IgG2a and IgG2b may boost the antibody response by binding to Fc-gamma receptors I and IV with high affinity. However, it remains to be tested if IgG1 and IgG3 are less effective than IgG2a and IgG2b in boosting immune responses by pre-formed complexes. In humans, all IgG subclasses, except IgG2, have been described to bind with high affinity to Fc-gamma receptor I (26).

The fact that injection of *in vitro* pre-formed IgG antigen immune complexes boosts the immune response implies a mechanism reminiscent of memory immune responses, where antigen-specific IgG is already present and forms immune complexes *in vivo* to accelerate the antigen-specific immune response. This is consistent with the known role of IgG in long-term immunity and its ability to form immune complexes that facilitate antigen presentation and T cell activation (21, 27, 28).

Different types of linkers are widely used in conjugate vaccines to enhance the efficacy of weakly immunogenic antigens by coupling them with carrier proteins (29). Theoretically, complexing antigens with IgG using a linker may direct immune responses toward the linker components. In fact, earlier studies addressing this question showed that immune responses might also be elicited against small molecule linkers (30). However, it is not clear whether this also applies to the maleimide linker used in our study or in anti-SARS-CoV-2 vaccines (31, 32). Notably, no significant induction of antibodies against the linker was detected in our experiments (**Supplementary Figure S5A**). Thus, although we cannot exclude that 1,2-PBM or any other linker may be targeted during immune responses, our data suggest that antibodies recognizing the linker are not increased in recall responses. Nevertheless, there are numerous different bis-maleimide linkers available, allowing for exchange or designing of optimized versions.

Given that IgG-containing immune complexes are part of normal physiological immune responses and the IgG used is a self-antigen, the induction of anti-IgG antibodies is not expected. In full agreement with our finding on the role of IgG in boosting immune responses, it has been reported that HIV-infected individuals failed to generate antigen-specific antibody responses unless they were also treated with a monoclonal IgG antibody targeting the antigen (33, 34). Together with our findings, these studies suggest that immune complexes comprised of antigen and IgG boost the humoral responses also in humans. Moreover, our data demonstrate that antigen specificity of the IgG is not required, as chemical cross-linking enables any antigen to be covalently linked to IgG (35). Consequently, even non-specific IgG conjugated to antigen is sufficient to elicit autologous antigen-specific immune responses.

The potential risk of autoreactive antibody responses following immunization was also addressed. We found increased autoantibody responses against protein C, particularly in individuals with high antigen-specific titers. Similar trends were observed in COVID-19 patients, suggesting a potential risk of autoreactivity with high antibody titers. This highlights the importance of monitoring autoreactive antibody responses in vaccine development and suggests that high antibody titers may be associated with an increased risk of autoreactivity. Such findings underscore the need for careful evaluation of immune responses to ensure that vaccines do not inadvertently trigger harmful autoimmunity (36, 37).

The study establishes two novel concepts highlighting the importance of (i) antigen activation and (ii) generating immune complexes *in vitro* prior to immunization. Antigens that can interact with the body’s immune system are set to activate immune responses and are therefore most suitable for vaccine design. In fact, activated RBD appears to enhance immune responses by forming complexes with other proteins *in vivo*, which may mimic the natural process of immune complex formation. This concept is supported by research indicating that enhanced antigen stability and presentation can significantly improve immunogenicity (18, 38). The data show that activated RBD binds to IgG *in vitro* depending on reactive maleimide groups (i. e. abolished in presence of free cysteine) and similarly, that this activated RBD can induce immune responses *in vivo*. This view is supported by the immune responses induced by pre-formed RBD*IgG complexes that were generated by concomitant incubation with 1,2-PBM. This suggests that antigen interaction with endogenous proteins, primarily immunoglobulins, is crucial for efficient immune responses and therefore vaccine design.

Cross-linking an antigen to IgG *in vitro* generates immune complexes that induce robust antigen-specific antibody responses. This *in vitro* cross-linking may mimic the natural memory response mechanism, where pre-existing antigen-specific IgG forms complexes that accelerate the immune response upon re-exposure to the antigen. This suggests that *in vitro* cross-linking could be a valuable strategy for boosting immune responses in vaccines. Studies have shown that immune complexes play a critical role in antigen presentation and the activation of B and T cells, highlighting the potential of this approach (19, 27, 28).

By using the SARS-CoV-2 RBD as a model antigen, this study provides valuable insights into the mechanisms of efficient and protective immune responses. The findings highlight the importance of both complex and monomeric antigens and underscore the potential of activated antigens and IgG cross-linking in vaccine design.

Together, our data suggest that pre-formed immune complexes can serve as a powerful tool in vaccine development. This approach has the potential to streamline the design of vaccines that are both efficient and effective at eliciting strong, protective immune responses possibly even in elderly people. These strategies could be applied to a wide range of pathogens, paving the way for the development of more effective vaccines. The ability to enhance immune responses through targeted antigen-modification and presentation strategies represents a promising avenue for future vaccine development. Thus, these findings offer a versatile and innovative platform for next-generation vaccine design, paving the way for more effective and widely applicable immunization strategies.

## Experimental procedures

### Mice

10–25 weeks old C57BL/6 wild-type mice were bred and housed in the animal facility of Ulm University under specific-pathogen-free conditions or purchased from Charles River. The majority of mice were female but also male mice were used in this study. Animal experiments were performed in compliance with licenses 1581 and 1653 for animal testing at the responsible regional board Tübingen, Germany. All animal experiments were done in compliance with the guidelines of the German law and were approved by the Animal Care and Use Committees of Ulm University and the local government.

### Human samples

Human plasma samples from COVID-19 positive individuals were obtained from T. Bakchoul via the CORE study “COVID-19 convalescence plasma from adult individuals”, ethics statement 897/2020BO2 (18.12.2020) and from a cohort of ICU COVID-19 patients, ethics statement 221/2020BO2 (02.04.2020).

### Cell culture and treatment with vaccines

Human embryonic kidney (HEK293T) cells were seeded in Iscove’s medium (Sigma-Aldrich) containing 10% heat-inactivated FCS (PAN-Biotech), 2 mM L-glutamine, 100 U/mL penicillin (Gibco), 100 U/mL streptomycin (Gibco), and 50 μM β-mercaptoethanol (Gibco) at a density of 0.25 x 10^6^ cells/mL per 6-well (Costar). For monitoring cell death and proliferation, HEK293T were seeded at a density of 0.025 x 10^6^ cells/mL per 96-well pre-coated with 0.01% poly-L-ornithine solution (Sigma-Aldrich). Treatment with the different vaccines was performed by adding 100 µL of Vaxzevria (AstraZeneca), Janssen (Johnson & Johnson), Spikevax (Moderna) or 60 µL of Comirnaty (BioNTech/Pfizer), respectively per 1 mL of culture medium, corresponding to 1/5^th^ of the human dose, dropwise to each well. For treatment with nRBD, 1,2-PBM (Santa Cruz), DMSO (Sigma-Aldrich), RBD* and RBD*IgG the concentrations of the respective compound in 1/5^th^ of the dose, which was applied in the mouse (~10 µg/mL) was calculated and added to the cells, respectively. Cell death and proliferation were analyzed by using the IncuCyte S3 platform (Sartorius) together with Cytotox Red reagent (Sartorius). Cells were incubated for 1 to 4 days before analysis as stated in the respective figure legends.

### HEK293-6E transfection and protein production

HEK293-6E (39) cells were maintained in F17 medium (Invitrogen) supplemented with 4 mM L-glutamine (Invitrogen), 0.1% Kolliphor P188 (Sigma-Aldrich), 25 μg/mL G418 (Invitrogen) and used for transfection during the exponential growth phase at a density of 1.5 - 2.0 x 10^6^ cells/mL and a viability of >97%. Sterile filtered Polyethylenimine (PEI, linear, 25 kDa, Polysciences) was added to pre-warmed (25 - 37˚C) F17 complete medium at a concentration of 2 µg/mL. In another tube, F17 complete medium was mixed with 1 µg/mL plasmid DNA. Contents of both tubes were vortexed, then mixed and subsequently pulse-vortexed for 3x 1 second. Following incubation for 3 min at room temperature (RT), the transfection mixture was added to the cell suspension.

Cells were incubated at 37˚C and 5% CO_2_, with agitation at 120 rpm (Eppendorf incubator New Brunswick S41i). After 24–48 hours (h), the cells were supplied with sterile filtered 20% w/v Tryptone N1 solution (Organotechnie) in complete F17 medium and harvested after 48 h for flow cytometric analysis. For generation of the soluble proteins nRBD and ProC, cells were transfected accordingly and the supernatant was harvested after 96–120 h.

### Protein purification

For production of RBD, an expression vector encoding a hexahistidine (His6)-tagged version of RBD (40) was kindly provided by F. Krammer & K. de la Rosa. The sequence for expression of His6-tagged human Protein C isoform 10 was cloned into a pTT5 expression vector backbone. Vectors were transiently transfected into HEK293-6E cells. Soluble native RBD and ProC were purified from the supernatant 5 days after transfection by nickel-based immobilized metal affinity chromatography (IMAC) (41). KLH was purchased from LGC Biosearch Technologies.

### Immunization

Mice were immunized intraperitoneally (i. p.) with 50 - 100 µg antigen in different configurations in a total volume of 100 µL PBS. If not stated otherwise, 50 µg CpG-ODN #1826 (Biomers) were added as adjuvant to primary and booster immunization mixtures. For primary immunizations with Alum, equal volumes of Alhydrogel 2%, (Invivogen) were added drop-wise to the antigens and subsequently incubated for 30 min at RT under agitation before injection. Mice immunized with Comirnaty (BioNTech/Pfizer) were i. p. immunized with 100 µl of the vaccine solution corresponding to one third of the dose administered in humans. Immunization was repeated as for RBD-complexes after 21 days. Pre-immune or PBS-immunized mice were considered as control-immunized (CI) animals.

### Antigen-modifications

RBD was biotinylated and complexed with streptavidin (SAV, Thermo Fisher Scientific) as described previously (7). RBD* was generated by addition of 20 µg 1,2 phenylen-bis-maleimide (1,2 PBM, Santa Cruz) per 100 µg native RBD and incubation over night at RT. 100 µg RBD* were quenched by addition of 1 µL of 2 M L-cysteine solution (Sigma-Aldrich) and 0,5 µL of 28% ammonium hydroxide solution to keep the pH at a neutral level. RBD*IgG, RBD*IgM, RBD*IgA were generated by mixing 100 µg native RBD with either 25 µg murine IgG (polyclonal), IgM (clone 11E10) or IgA (clone S107) all purchased from Southern Biotech, respectively. RBD*IgG*IgM and RBD*IgG*IgM*IgA were generated by adding 25 µg from each of the respective immunoglobulins to 100 µg RBD. RBD*Ig complexes were subsequently generated as previously described for RBD*. If stated in the figure legend, dialysis was performed by using 10K cut-off columns (Amicon Ultra, Merck Millipore) according the manufacturer’s instructions.

### Coomassie staining and Immunoblot assay

Equal amounts of proteins or cell lysates were used directly or denatured in presence of β-mercaptoethanol (2-ME, Sigma-Aldrich) and separated by SDS-gel electrophoresis on a 10% gel. The gel was either stained with Coomassie solution (Coomassie Brilliant Blue R-250 (0.025% w/v, Bio-Rad), methanol (50% v/v, Sigma-Aldrich), glacial acetic acid (10% v/v, Fisher Chemical) and destained with methanol (30% v/v), glacial acetic acid (20% v/v) or transferred onto a nitrocellulose membrane for immunoblotting. The membrane was blocked for 1 h with TBS-T (TBS/0.1% Tween (Sigma-Aldrich)) containing 5% bovine serum albumin (BSA, Serva). Serum from immunized mice diluted 1: 50, rabbit α/β-tubulin antibody (Cell Signaling Technology) diluted 1: 1000 and anti-Ig kappa light chain antibody (Southern Biotech) diluted 1: 5000 in TBS-T supplemented with 5% milk powder (Fluka) were used as primary antibodies for overnight probing of the membrane.

After three washing steps in TBS-T (15 min each), the membrane was incubated for 1 h at RT with horseradish peroxidase (HRP)-coupled secondary goat α-rabbit, α-goat or α-mouse antibodies (Pierce, Thermo Fisher Scientific) diluted in TBS-T/2% BSA.

Antibody in excess was washed off and stained proteins were detected with ECL Ultra Solution kit (Lumigen) on a Fusion SL advanced imaging system (Vilber Lourmat).

### Immunoprecipitation of RBD*IgG complexes and dot blot

nRBD was activated by 1,2-PBM treatment and subsequent incubation for 1 h at RT. The mixture was then dialyzed against PBS using a 10 kDa cut-off column (Amicon Ultra, Merck Millipore). The resulting sample, containing RBD*, was split and mixed either directly with IgG at a molar ratio of 10: 1 or after quenching with 1 M cysteine solution as a control, followed by overnight incubation at RT. Additionally, another sample was prepared by activating RBD with 1,2-PBM in presence of IgG directly, as described in the “Antigen Modifications” section. All samples were precipitated using Protein G Sepharose (GE Healthcare, 17-0618-01), washed with 10 volumes of PBS-T, and eluted in 0.1 M glycine-HCl (pH 2.7, Sigma-Aldrich). The pH of the eluates was immediately adjusted with an appropriate volume of neutralization buffer (1 M Tris-HCl, pH 9, Sigma-Aldrich). The eluates were then applied in 2 µL droplets to a nitrocellulose membrane and allowed to dry for at least 30 min. The membrane was subsequently blocked in TBS-T containing 5% BSA and processed as a western blot membrane, as described in the “Coomassie Staining and Immunoblot Assay” section.

For detection of complexes with different Ig isotypes, RBD was coupled with NHS-activated agarose beads (+Cytiva), mixed with IgA (clone S107), IgE (clone 15.3), total IgG (polyclonal), IgG1 (clone 15H6) or IgG2b (clone A-1) all purchased from Southern Biotech and complexed with 1,2-PBM. Samples were washed, denatured for 30 min at 95°C in presence of 2-ME and subjected to immunoblotting as described in the “Coomassie Staining and Immunoblot Assay” section.

### Enzyme-linked immunosorbent assay

96-well plates (NUNC, maxisorp) were coated either with 10 µg/mL nRBD, SAV (Sigma-Aldrich), polyclonal mouse IgG (Southern Biothech), BSA mixed with 20 µg 1,2-PBM per 100 µg protein, or 2.5 µg/mL Protein C, respectively. Coating with polyclonal anti-murine or -human IgM or -IgG (Southern Biothech), respectively, was used as standard when measuring anti-mouse RBD, SAV, anti-1,2-PBM or anti-IgG IgM antibodies. The plates were subsequently blocked with buffer containing 1% BSA. Dilutions of murine IgM (clone: 11E10, Southern Biotech) or polyclonal IgG antibodies (Southern Biotech) were used as standards for determining anti-RBD, -SAV, -1,2-PBM or -IgG antibodies. A human serum sample from a vaccinated donor served as reference for measuring anti-human RBD levels, while a mouse anti-Protein C antibody (Santa Cruz) was used as standard to quantify anti-Protein C concentrations. For detection of anti-SARS-CoV-2 antibodies in the sera of RBD-immunized mice as well as convalescent and vaccinated humans the samples were pre-diluted 1: 200. For detection of anti-Protein C antibodies, the sera were pre-diluted 1: 10. For detection of anti-1,2-PBM and -IgG IgM antibodies a pre-dilution of 1: 50 was used. Pre-diluted samples were applied as duplicates in dilution steps of 1: 3 to the coated plates. The concentration of IgM or IgG antibodies in the sera was determined by detection with alkaline phosphatase-labeled anti-mouse or human IgM or IgG (Southern Biotech), respectively. IL-6 concentrations were determined by using a commercial ELISA kit (ENZO, ADI-900-045) according to the manufacturer's instructions. The substrate p-nitrophenylphosphate (Genaxxon) in diethanolamine-buffer was added and data were acquired at 405 nm using a Multiskan FC ELISA plate reader (Thermo Scientific).

### Neutralization assay

Caco-2 cells were seeded in 180 µL at a density of 0.01 x 10^6^ cells/mL. After one day of incubation, heat-inactivated sera were serially diluted in PBS and mixed with SARS-CoV-2 pseudoviral particles (PP) at a ratio of 1: 1 to the cells with 10% serum at a maximum. Serum and SARS-CoV-2 PP were mixed for 1 h at 37°C and subsequently added to Caco-2 cells in duplicates at a 1: 10 dilution (max. 1% serum on cells) and incubated cells at 37°C. 16 h post-transduction, Firefly luciferase activity was measured by using the Promega Luciferase Assay System (E1501), and values were normalized to PPs treated with PBS only. The EC50 was calculated by applying the IC50 fit by Prism Inhibitor vs. Normalized response (variable slope).

### Flow cytometry

Cell suspensions were treated with Fc-Block antibodies and stained by standard procedures. Viable cells were distinguished from dead cells by staining with Fixable Viability Dye eFluor 450 or 780 (eBioscience, Thermo Fisher Scientific). Total serum from RBD- or BNT-immunized mice was used to stain SARS-CoV-2 spike protein, expressed on the surface of human cells and detected by subsequent staining with anti-mouse IgG antibody (APC, clone Poly4053, BioLegend). Antigen-specific germinal center B cells and plasma cells were detected by staining with biotinylated RBD detected with SAV-Alexa Fluor 647 (Jackson ImmunoResearch) and antibodies detecting CD19 (FITC, clone 6D5, BioLegend), B220 (PerCP-eFluor 710, clone RA3-6B2, eBioscience, Thermo Fisher Scientific), CD38 (BV510, BD Biosciences), CD95 (PE-Cy7, clone Jo2, BD Biosciences), anti-mouse T- and B-cell activation antigen (BV421, clone GL7, BD Biosciences) and CD138 (PE, clone 281-2, BD Biosciences). Cells were acquired at a FACS Canto II or FACS LSR Fortessa flow cytometer (BD Biosciences) or a CytoFLEX S (Beckman Coulter) and analyzed in FlowJo version 10 software (TreeStar).

### Statistical analysis

Graphs were created and statistical analysis was performed by using GraphPad Prism (Version 10) software. The numbers of individual replicates or mice (n) is stated in the figure legends as well as the tests applied to calculate statistical significance among observed differences. P values < 0.05 were considered to be statistically significant (n. s. = not significant; *p ≤ 0.05; **p ≤ 0.01; ***p ≤ 0.001; ****p ≤ 0.0001).

## Data Availability

The original contributions presented in the study are included in the article/**Supplementary Material**. Further inquiries can be directed to the corresponding authors.

## References

[1] Plotkin SA. Correlates of protection induced by vaccination. Clin Vaccine Immunol. (2010) 17:1055–65. doi: 10.1128/CVI.00131-10 PMC2897268 20463105

[2] Kato Y Abbott RK Freeman BL Haupt S Groschel B Silva M. Multifaceted effects of antigen valency on B cell response composition and differentiation *in vivo* . Immunity. (2020) 53:548–563 e8. doi: 10.1016/j.immuni.2020.08.001 32857950 PMC7451196

[3] Ferapontov A Omer M Baudrexel I Nielsen JS Dupont DM Juul-Madsen K. Antigen footprint governs activation of the B cell receptor. Nat Commun. (2023) 14:976. doi: 10.1038/s41467-023-36672-0 36813795 PMC9947222

[4] Victora GD Nussenzweig MC. Germinal centers. Annu Rev Immunol. (2012) 30:429–57. doi: 10.1146/annurev-immunol-020711-075032 22224772

[5] Roes J Rajewsky K Immunoglobulin D. (IgD)-deficient mice reveal an auxiliary receptor function for IgD in antigen-mediated recruitment of B cells. J Exp Med. (1993) 177:45–55. doi: 10.1084/jem.177.1.45 8418208 PMC2190865

[6] Setz CS Khadour A Renna V Iype J Gentner E He X. Pten controls B-cell responsiveness and germinal center reaction by regulating the expression of IgD BCR. EMBO J. (2019) 38(11):e100249. doi: 10.15252/embj.2018100249 31015337 PMC6545559

[7] Ubelhart R Hug E Bach MP Wossning T Duhren-von Minden M Horn AH. Responsiveness of B cells is regulated by the hinge region of IgD. Nat Immunol. (2015) 16:534–43. doi: 10.1038/ni.3141 25848865

[8] Racine R Winslow GM. IgM in microbial infections: taken for granted? Immunol Lett. (2009) 125:79–85. doi: 10.1016/j.imlet.2009.06.003 19539648 PMC2747358

[9] Ossendorp F Ho NI Van Montfoort N. How B cells drive T-cell responses: A key role for cross-presentation of antibody-targeted antigens. Adv Immunol. (2023) 160:37–57. doi: 10.1016/bs.ai.2023.09.002 38042585

[10] To KK Sridhar S Chiu KH Hung DL Li X Hung IF. Lessons learned 1 year after SARS-CoV-2 emergence leading to COVID-19 pandemic. Emerg Microbes Infect. (2021) 10:507–35. doi: 10.1080/22221751.2021.1898291 PMC8006950 33666147

[11] Jackson CB Farzan M Chen B Choe H. Mechanisms of SARS-CoV-2 entry into cells. Nat Rev Mol Cell Biol. (2022) 23:3–20. doi: 10.1038/s41580-021-00418-x 34611326 PMC8491763

[12] Renn A Fu Y Hu X Hall MD Simeonov A. Fruitful neutralizing antibody pipeline brings hope to defeat SARS-cov-2. Trends Pharmacol Sci. (2020) 41:815–29. doi: 10.1016/j.tips.2020.07.004 PMC7572790 32829936

[13] Robotham AC Kelly JF. Detection and quantification of free sulfhydryls in monoclonal antibodies using maleimide labeling and mass spectrometry. MAbs. (2019) 11:757–66. doi: 10.1080/19420862.2019.1595307 PMC6601545 30894096

[14] Walker FJ Fay PJ. Regulation of blood coagulation by the protein C system. FASEB J. (1992) 6:2561–7. doi: 10.1096/fasebj.6.8.1317308 1317308

[15] Sarangi PP Lee HW Kim M. Activated protein C action in inflammation. Br J Haematol. (2010) 148:817–33. doi: 10.1111/j.1365-2141.2009.08020.x PMC2868910 19995397

[16] Esmon CT. Inflammation and the activated protein C anticoagulant pathway. Semin Thromb Hemost. (2006) 32 Suppl 1:49–60. doi: 10.1055/s-2006-939554 16673266

[17] Tai WB He L Zhang XJ Pu J Voronin D Jiang SB. Characterization of the receptor-binding domain (RBD) of 2019 novel coronavirus: implication for development of RBD protein as a viral attachment inhibitor and vaccine. Cell Mol Immunol. (2020) 17:613–20. doi: 10.1038/s41423-020-0400-4 PMC7091888 32203189

[18] Martin GM Russell RA Mundsperger P Harris S Jovanoska L Trajano LF. Profound structural conservation of chemically cross-linked HIV-1 envelope glycoprotein experimental vaccine antigens. NPJ Vaccines. (2023) 8:101. doi: 10.1038/s41541-023-00696-w 37443366 PMC10345191

[19] Kim YM Pang JY Korbel GA Peperzak V Boes M Ploegh HL. Monovalent ligation of the B cell receptor induces receptor activation but fails to promote antigen presentation. Proc Natl Acad Sci U S A. (2006) 103(9):3327–32. doi: 10.1073/pnas.0511315103 PMC1413933 16492756

[20] Lu LL Suscovich TJ Fortune SM Alter G. Beyond binding: antibody effector functions in infectious diseases. Nat Rev Immunol. (2018) 18:46–61. doi: 10.1038/nri.2017.106 29063907 PMC6369690

[21] Junker F Gordon J Qureshi O. Fc gamma receptors and their role in antigen uptake, presentation, and T cell activation. Front Immunol. (2020) 11:1393. doi: 10.3389/fimmu.2020.01393 32719679 PMC7350606

[22] Nimmerjahn F Ravetch JV. Fcgamma receptors as regulators of immune responses. Nat Rev Immunol. (2008) 8:34–47. doi: 10.1038/nri2206 18064051

[23] Takai T. Roles of Fc receptors in autoimmunity. Nat Rev Immunol. (2002) 2:580–92. doi: 10.1038/nri856 12154377

[24] Mora N Rosales C. Fc receptor functions in host and immune regulation. Rev Invest Clin. (2009) 61:313–26. 19848309

[25] Shibuya A Honda SI Shibuya K. A pro-inflammatory role of Fcalpha/muR on marginal zone B cells in sepsis. Int Immunol. (2017) 29:519–24. doi: 10.1093/intimm/dxx059 29281010

[26] Bruhns P. Properties of mouse and human IgG receptors and their contribution to disease models. Blood. (2012) 119:5640–9. doi: 10.1182/blood-2012-01-380121 22535666

[27] Schmidt F Weisblum Y Muecksch F Hoffmann HH Michailidis E Lorenzi JCC. Measuring SARS-CoV-2 neutralizing antibody activity using pseudotyped and chimeric viruses. J Exp Med. (2020) 217(11):e20201181. doi: 10.1084/jem.20201181 32692348 PMC7372514

[28] Stadlbauer D Amanat F Chromikova V Jiang K Strohmeier S Arunkumar GA. SARS-coV-2 seroconversion in humans: A detailed protocol for a serological assay, antigen production, and test setup. Curr Protoc Microbiol. (2020) 57:e100. doi: 10.1002/cpmc.v57.1 32302069 PMC7235504

[29] Timmerman JM. Carrier protein conjugate vaccines: the "missing link" to improved antibody and CTL responses? Hum Vaccin. (2009) 5:181–3. doi: 10.4161/hv.5.3.7476 19246995

[30] Boeckler C Frisch B Muller S Schuber F. Immunogenicity of new heterobifunctional cross-linking reagents used in the conjugation of synthetic peptides to liposomes. J Immunol Methods. (1996) 191:1–10. doi: 10.1016/0022-1759(95)00284-7 8642195

[31] Eugenia-Toledo-Romani M Verdecia-Sanchez L Rodriguez-Gonzalez M Rodriguez-Noda L Valenzuela-Silva C Paredes-Moreno B. Safety and immunogenicity of anti-SARS CoV-2 vaccine SOBERANA 02 in homologous or heterologous scheme: Open label phase I and phase IIa clinical trials. Vaccine. (2022) 40:4220–30. doi: 10.1016/j.vaccine.2022.05.082 PMC9167831 35691871

[32] Valdes-Balbin Y Santana-Mederos D Quintero L Fernandez S Rodriguez L Sanchez Ramirez B. SARS-coV-2 RBD-tetanus toxoid conjugate vaccine induces a strong neutralizing immunity in preclinical studies. ACS Chem Biol. (2021) 16:1223–33. doi: 10.1021/acschembio.1c00272 34219448

[33] Schoofs T Klein F Braunschweig M Kreider EF Feldmann A Nogueira L. HIV-1 therapy with monoclonal antibody 3BNC117 elicits host immune responses against HIV-1. Science. (2016) 352:997–1001. doi: 10.1126/science.aaf0972 27199429 PMC5151174

[34] Gunst JD Pahus MH Rosas-Umbert M Lu IN Benfield T Nielsen H. Early intervention with 3BNC117 and romidepsin at antiretroviral treatment initiation in people with HIV-1: a phase 1b/2a, randomized trial. Nat Med. (2022) 28:2424–35. doi: 10.1038/s41591-022-02023-7 PMC10189540 36253609

[35] Dvorscek AR McKenzie CI Staheli VC Ding Z White J Fabb SA. Conversion of vaccines from low to high immunogenicity by antibodies with epitope complementarity. Immunity. (2024) 57:2433–2452 e7. doi: 10.1016/j.immuni.2024.08.017 39305904

[36] Gruber CN Patel RS Trachtman R Lepow L Amanat F Krammer F. Mapping systemic inflammation and antibody responses in multisystem inflammatory syndrome in children (MIS-C). Cell. (2020) 183:982–995 e14. doi: 10.1016/j.cell.2020.09.034 32991843 PMC7489877

[37] Addetia A Crawford KH Dingens A Zhu H Roychoudhury P Huang ML. Neutralizing antibodies correlate with protection from SARS-CoV-2 in humans during a fishery vessel outbreak with high attack rate. medRxiv. (2020) 58(11):e02107-20. doi: 10.1101/2020.08.13.20173161 PMC7587101 32826322

[38] Scheiblhofer S Laimer J MaChado Y Weiss R Thalhamer J. Influence of protein fold stability on immunogenicity and its implications for vaccine design. Expert Rev Vaccines. (2017) 16:479–89. doi: 10.1080/14760584.2017.1306441 PMC5490637 28290225

[39] Durocher Y Perret S Kamen A. High-level and high-throughput recombinant protein production by transient transfection of suspension-growing human 293-EBNA1 cells. Nucleic Acids Res. (2002) 30:E9. doi: 10.1093/nar/30.2.e9 11788735 PMC99848

[40] Amanat F Stadlbauer D Strohmeier S Nguyen THO Chromikova V McMahon M. A serological assay to detect SARS-CoV-2 seroconversion in humans. medRxiv. (2020) 26(7):1033–1036. doi: 10.1101/2020.03.17.20037713 PMC8183627 32398876

[41] Kielkopf CL Bauer W Urbatsch IL. Purification of polyhistidine-tagged proteins by immobilized metal affinity chromatography. Cold Spring Harb Protoc. (2020) 2020:102194. doi: 10.1101/pdb.prot102194 32482902

