# Supplementary material for: Mimicking immune complexes for efficient antibody responses
Source: Front Immunol. 2025 Apr 28;16:1570487. doi: 10.3389/fimmu.2025.1570487 (PMC12066251; doi:10.3389/fimmu.2025.1570487)
Supplement: Supplementary file 1 [file DataSheet1.pdf]

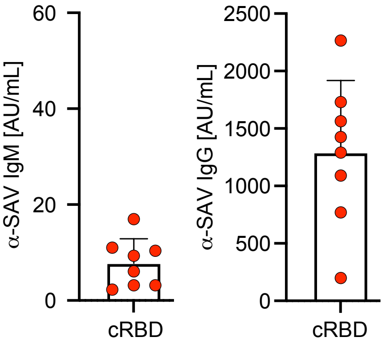

Supplementary Figure S1 | Anti-RBD serum used in neutralization assay and anti-Streptavidin (SAV) response Concentrations of SAV-specific IgM and IgG were determined by ELISA (n = 7) in sera from **Figure 1F**, collected at day 28 one week after booster vaccination with SAV-complexed RBD. n = 7, mean ± SD.

Figure S1 | Anti-SAV response

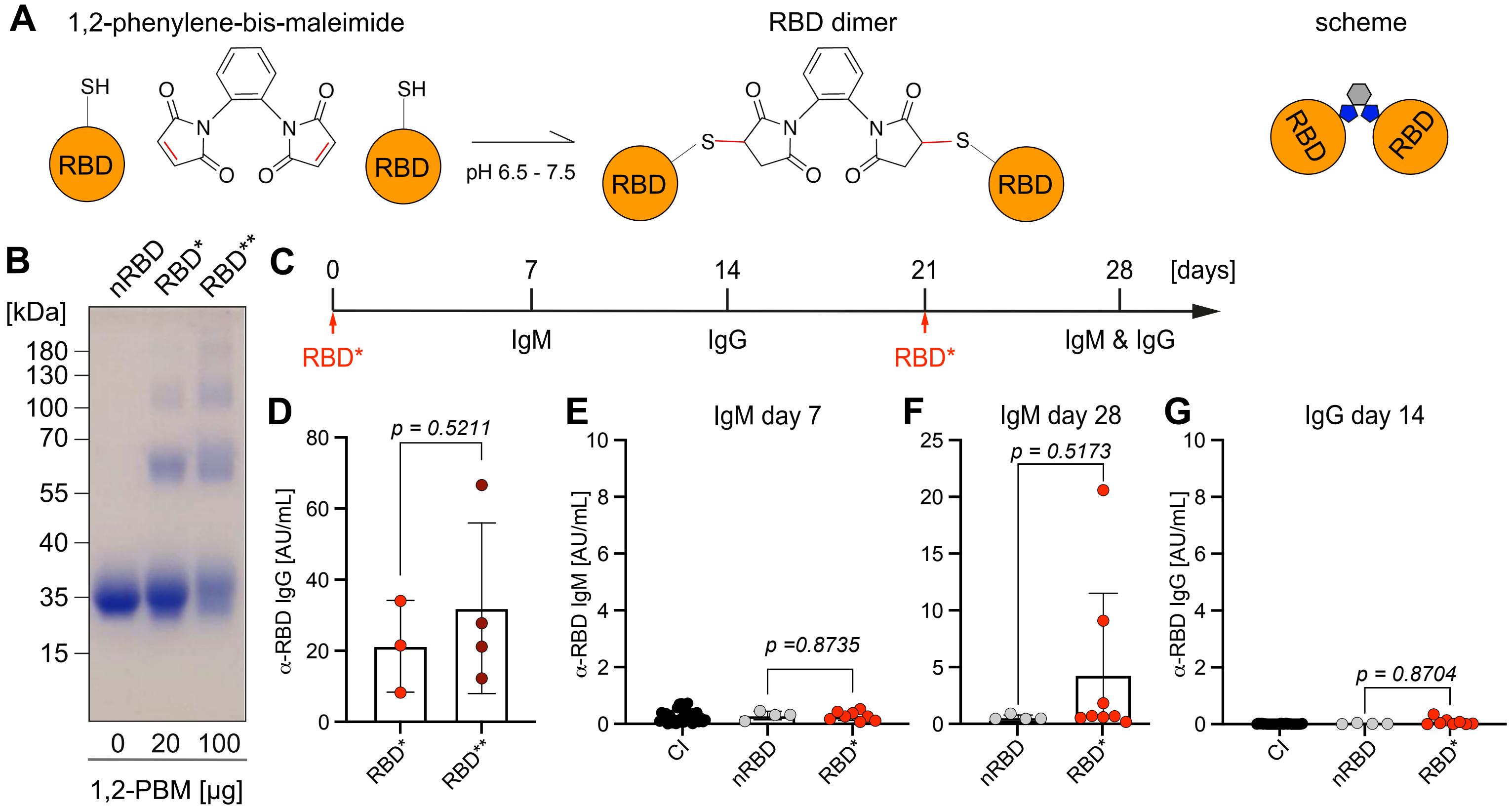

Supplementary Figure S2 | Activated RBD is required for enhanced immune responses
A | Reaction scheme of chemical cross-linking. At pH 6.5 - 7.5 reactive groups (marked in red) of 1,2-phenylene-bis-maleimide (1,2-PBM) undergo oxidation with sulfhydryl (SH)-groups on cysteine residues of proteins, here the receptor-binding domain (RBD) of the severe acute respiratory syndrome coronavirus 2 (SARS-CoV-2) spike protein, to form a stable thioether linkage. **B |** 100 µg of native (n)RBD (~27kDa) were complexed by addition of 20 µg 1,2-PBM for generation of RBD*, while a 5-fold higher amount (100 µg) of 1,2-PBM was used to generate RBD**. Samples were separated under reducing conditions on a 10% SDS-gel and detected by Coomassie-staining.
C | Schematic overview of immunization procedure: C57BL/6 WT mice were immunized intraperitoneally (i. p.) with 50 µg of RBD* in presence of CpG-ODN #1826 as adjuvant. Immunization was repeated in the same way on day 21. Serum samples were collected on days 7, 14 and after booster vaccination on day 28 and RBD-specific IgM and IgG concentrations were analyzed by ELISA.
D | Mice were immunized as described in **C** with 50 µg of RBD* (n = 3) or RBD** (n = 4) in presence of CpG-ODN #1826. Immunization was repeated in the same way on day 21. RBD-specific IgG levels were determined by ELISA after booster vaccination on day 28. Mean ± SD, statistical significance was calculated by using the unpaired t test.
E – G | Serum was collected from mice immunized with nRBD (n = 7) and RBD* (n = 11), as illustrated in **C** on day 28. RBD-specific antibody concentrations were measured by ELISA and compared to titers measured in CI mice (n = 29) for IgM on day 7 (**E**) and 28 (**F**) and for IgG on day 14 (**G**), respectively. Mean ± SD, statistical significance was calculated by applying the ordinary one-way ANOVA for **E**, and the Kruskal-Wallis test for **F** and **G**.

Figure S2 | Activated RBD is required for enhanced immune responses

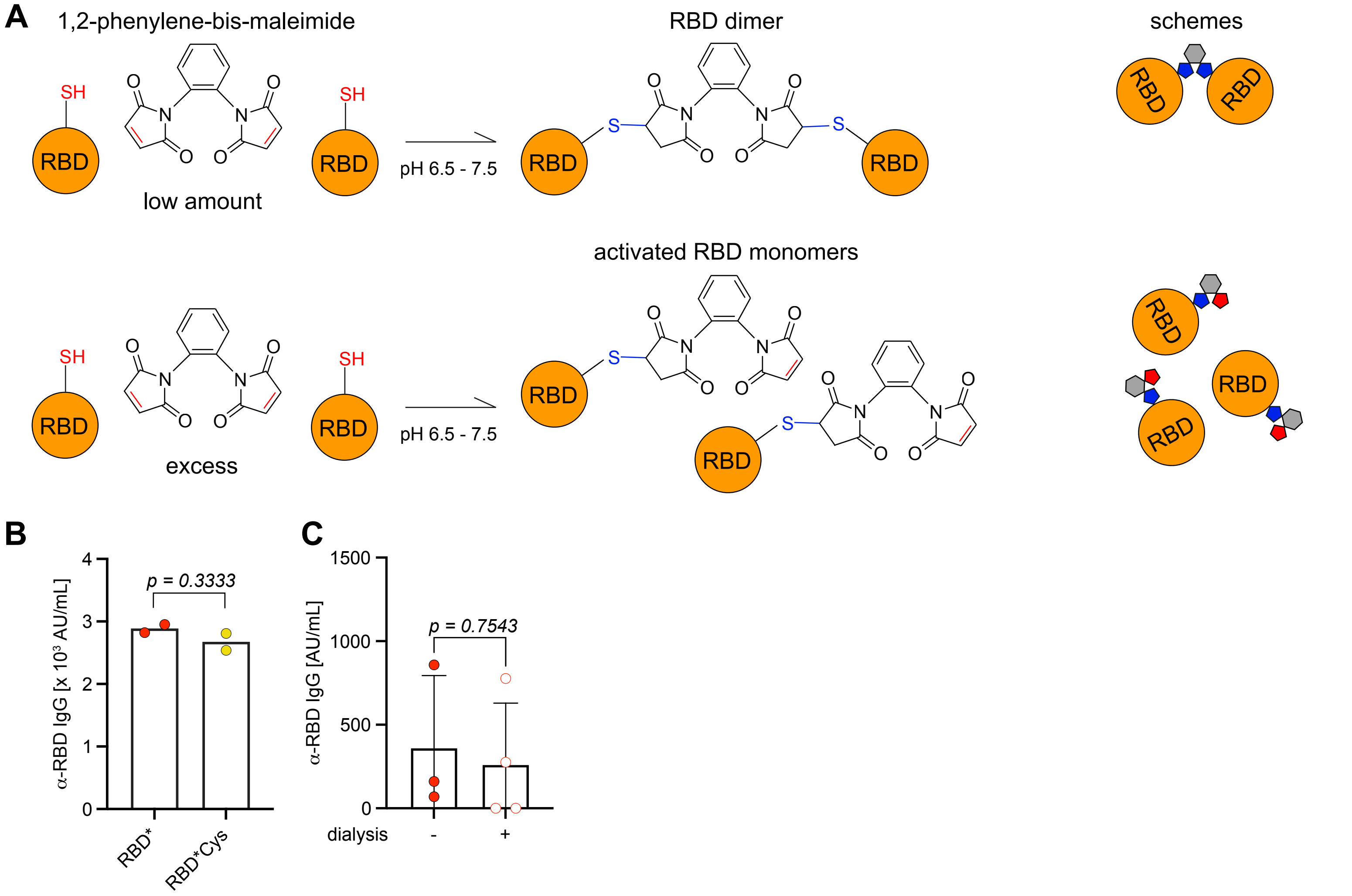

Supplementary Figure S3 | Schematic concept of activated antigen

A | Schematic concept of activated antigen: Low amounts of 1,2-PBM lead to formation of RBD dimers, while excess of 1,2-PBM generates activated RBD monomers in which a free maleimide group is still available for reaction with SH-groups in other proteins.

B | Comparable concentrations of activated RBD (RBD*; n = 2) and cysteine-quenched RBD*Cys (RBD*Cys; n = 2) were coated and detected by ELISA with serum containing anti-RBD-specific IgG. Mean ± SD, statistical significance was calculated by applying the Mann-Whitney-U test.

C | Mice were immunized as described in **Supplementary Figure S2C** with 50 µg of RBD* either subjected to dialysis prior to injection to remove excess 1,2-PBM (n = 4) or left undialysed (n = 3). Immunization was repeated in the same way on day 21. RBD-specific IgG levels were determined by ELISA after booster vaccination on day 28. Mean ± SD, statistical significance was calculated by using the unpaired t test.

Figure S3 | Schematic concept of activated antigens

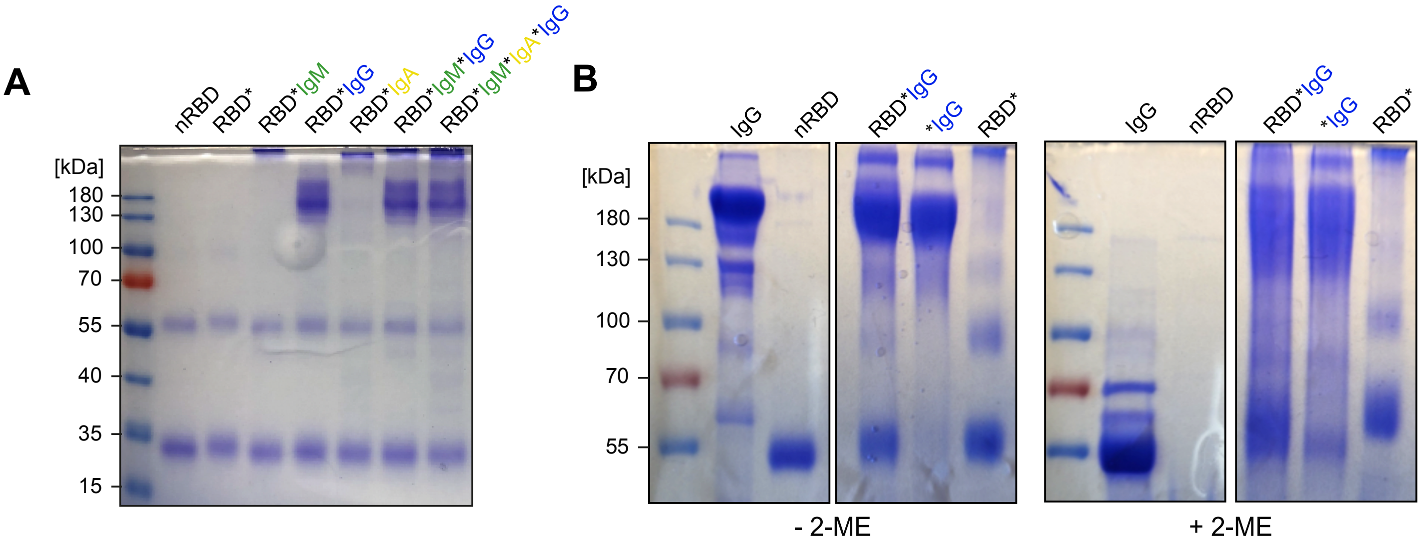

C

no.	group
#1<br>#2<br>#3	nRBD
#4<br>#5<br>#6	RBD*
#7<br>#8<br>#9	RBD*IgM
#10<br>#11<br>#12	RBD*IgG
#13<br>#14<br>#15	RBD*IgA
#16<br>#17<br>#18	RBD*IgG*IgM
#19<br>#20<br>#21	RBD*IgG*IgM*IgA

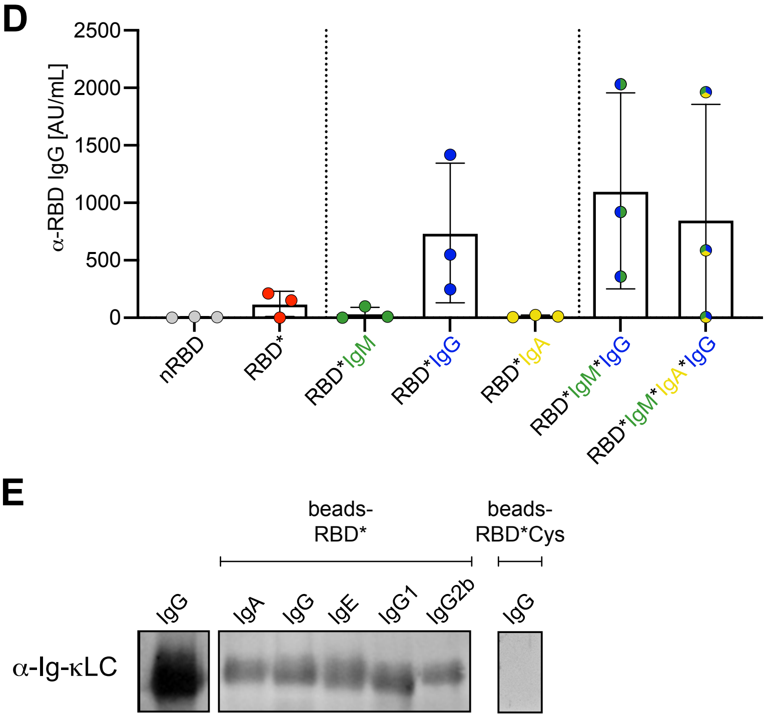

Supplementary Figure S4 | IgG is required for efficient immune responses by RBD*

A | Different antigen complexes were generated by treating nRBD with 1, 2-PBM in presence of either 25 µg murine IgM, IgG or IgA immunoglobulins, respectively. In addition, complexes comprising combinations of IgM and IgG (RBD*IgM*IgG) or all three isotypes (RBD*IgM*IgA*IgG) were generated. RBD*Ig complexes were compared to nRBD and RBD* by separation on a 10% SDS-PAGE under non-reducing conditions and subsequent Coomassie-staining.

B | Complexes were generated by treating nRBD with 1, 2-PBM in presence of 25 µg IgG immunoglobulins, respectively. RBD*IgG complexes were compared to nRBD, RBD*, IgG and *IgG by separation on a 10% SDS-PAGE under non-reducing (-2-ME) and reducing (+2-ME) conditions and subsequent Coomassie-staining.

C | Tabular overview of mouse groups in an immunization experiment, testing RBD* complexes with different immunoglobulin isotypes.

D | Immunization was performed in WT mice with nRBD, RBD* and RBD*Ig complexes (shown in**Supplementary Figure S4A**) according to the procedure described in **Supplementary Figure S2C** in presence of 50 µg CpG-ODN #1826 as adjuvant. Serum was collected on day 28, one week after secondary immunization and RBD-specific IgG concentrations were measured by ELISA. N = 3, mean ± SD.

E | Immunoblot analysis for presence of Ig-κ light chain (LC) in RBD complexed with different Ig isotypes. NHS-activated agarose beads were coupled with RBD and subjected to 1,2-PBM-mediated activation in presence of either IgA, IgG, IgE, IgG1 or IgG2b. Beads coupled with cysteine-inactivated RBD* (RBD*Cys) that were subsequently incubated with IgG served as controls. After washing and sample preparation, the resulting protein-complexes were subjected to immunoblotting together with IgG as a positive control and probed with anti-Ig-κLC antibodies.

Figure S4 | IgG is required for efficient immune responses by RBD*

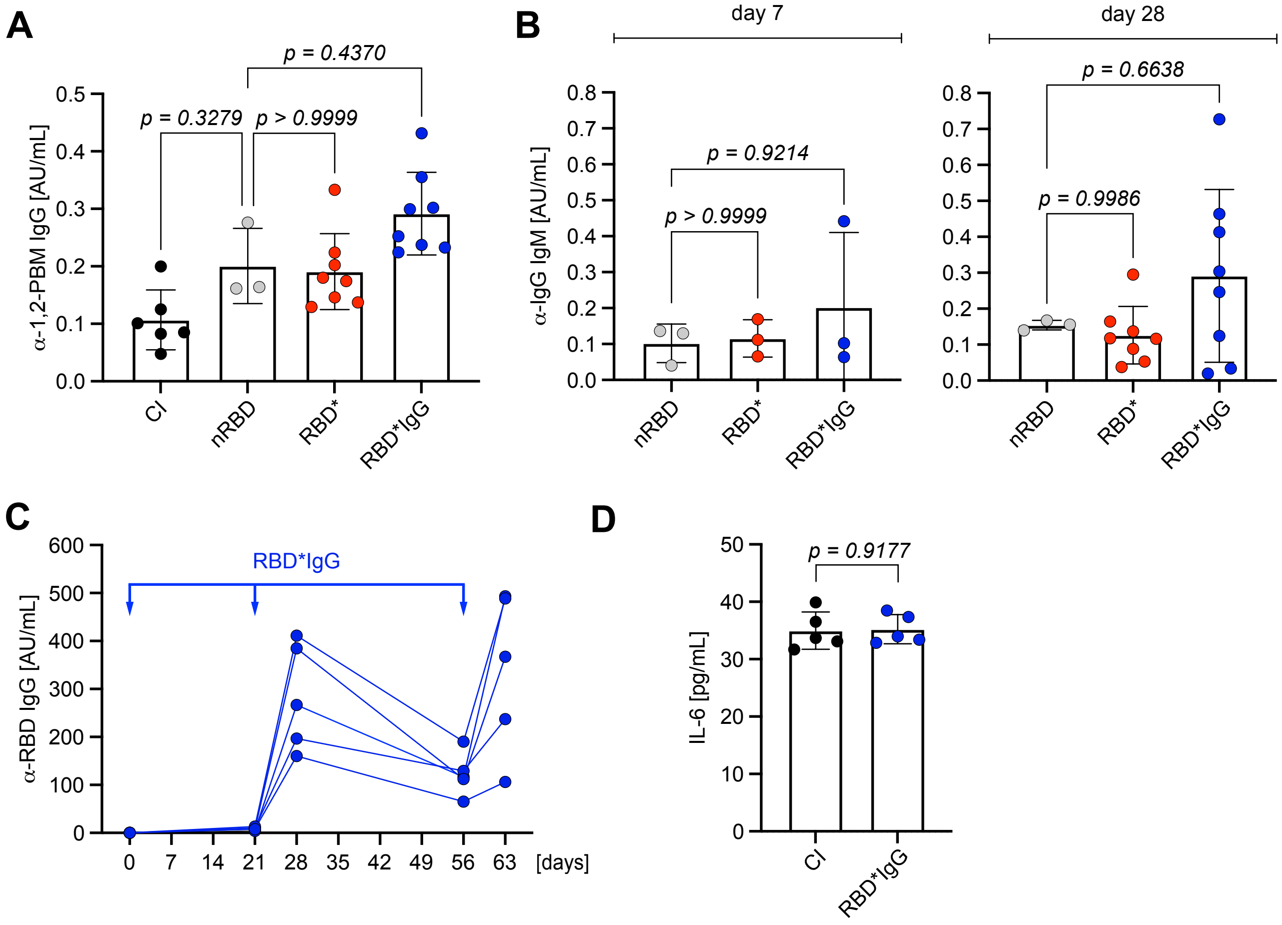

Supplementary Figure S5 | Antibodies against immune complex components

A | Anti-1,2-PBM IgG titers measured by ELISA in serum collected on day 28 from CI-mice (n = 5), mice immunized either with nRBD (n = 3), RBD* (n = 8) or RBD*IgG (n = 8), mean ± SD. Blocking buffer containing BSA was mixed with 1,2-PBM (20 µg per 100 µg BSA) and used for coating. Statistical significance was calculated by applying the Kruskal-Wallis test.

B | Anti-IgG IgM titers measured by ELISA in serum collected on day 7 (n = 3 for all groups) and 28 from mice immunized either with nRBD (n = 3), RBD* (n = 8) or RBD*IgG (n = 8), mean ± SD. Statistical significance was calculated by applying the ordinary one-way ANOVA.

C | Anti-RBD IgG levels in sera from RBD*IgG-immunized mice (n = 5) measured by ELISA on days 21, 28, 56 and 63. Mice were immunized as described in **Supplementary Figure S2C** with 50 µg RBD*IgG on days 0, 21 and 56.

D | IL-6 concentration measured by ELISA in serum from CI-mice or mice immunized with RBD*IgG collected on day 28, (n = 5) mean ± SD. Statistical significance was calculated by applying the paired t test.

Figure S5 | Antibodies against immune complex components

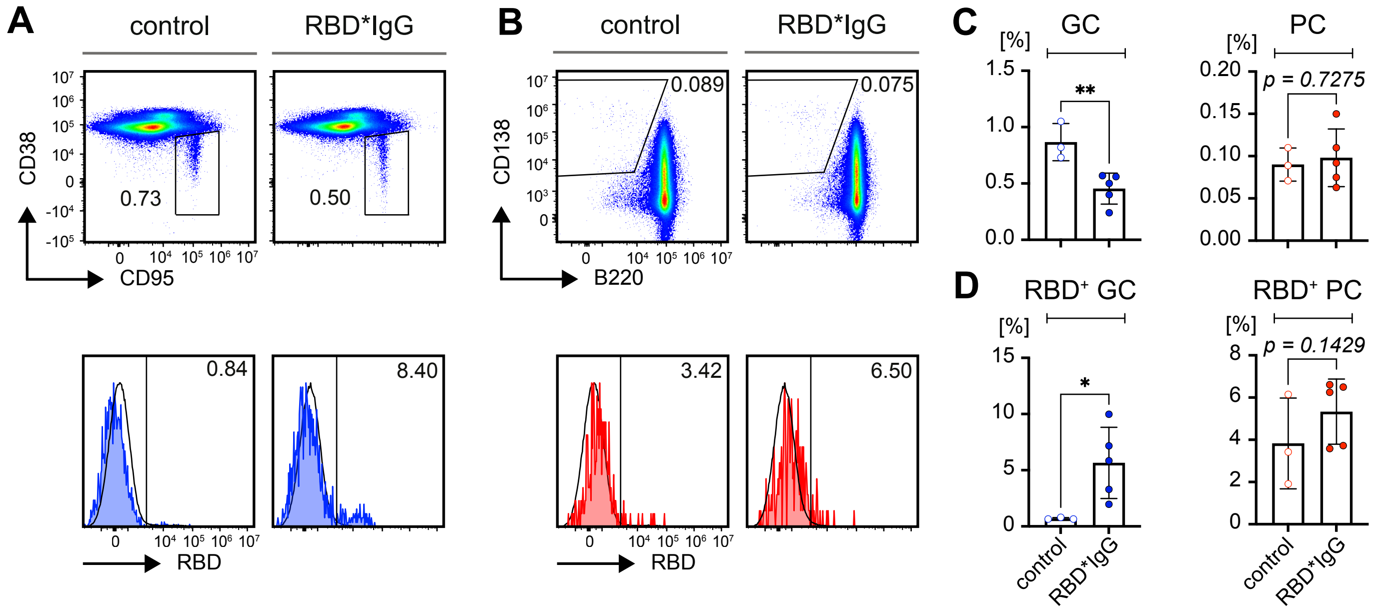

Supplementary Figure S6 | RBD-specific germinal center B and plasma cells

Mice were immunized as described in **Supplementary Figure S2C** with 50 µg RBD*IgG on days 0, 21 and 42 (n = 5). Spleens were harvested and stained for RBD-specific germinal center (GC) B cells and plasma cells (PC). Unimmunized mice served as controls (n = 3).

A | Representative flow cytometric analysis of RBD-recognition by GC B cells. GC B cells were identified by gating on $CD19^+/CD38^-/CD95^+$ and $GL7^+$ cells. The antigen-binding capacity was assessed by staining with biotinylated RBD.

B | Representative flow cytometric analysis of RBD-recognition by plasma cells. Plasma cells were identified by gating on $CD19^+/B220^{lo}/CD138^+$ cells. The antigen-binding capacity was assessed by staining with biotinylated RBD.

C | Quantified percentages of total GC B (left panel) and plasma cells (right panel) in spleens from RBD*IgG-immunized mice and controls. Statistical significance was calculated by applying the unpaired t test.

D | Quantified percentages of RBD-specific GC B (left panel) and PCs (right panel) in spleens from RBD*IgG-immunized mice and controls. Statistical significance was calculated by applying the unpaired t test or the Mann-Whitney-U test, respectively.

Figure S6 | RBD-specific germinal center B and plasma cells

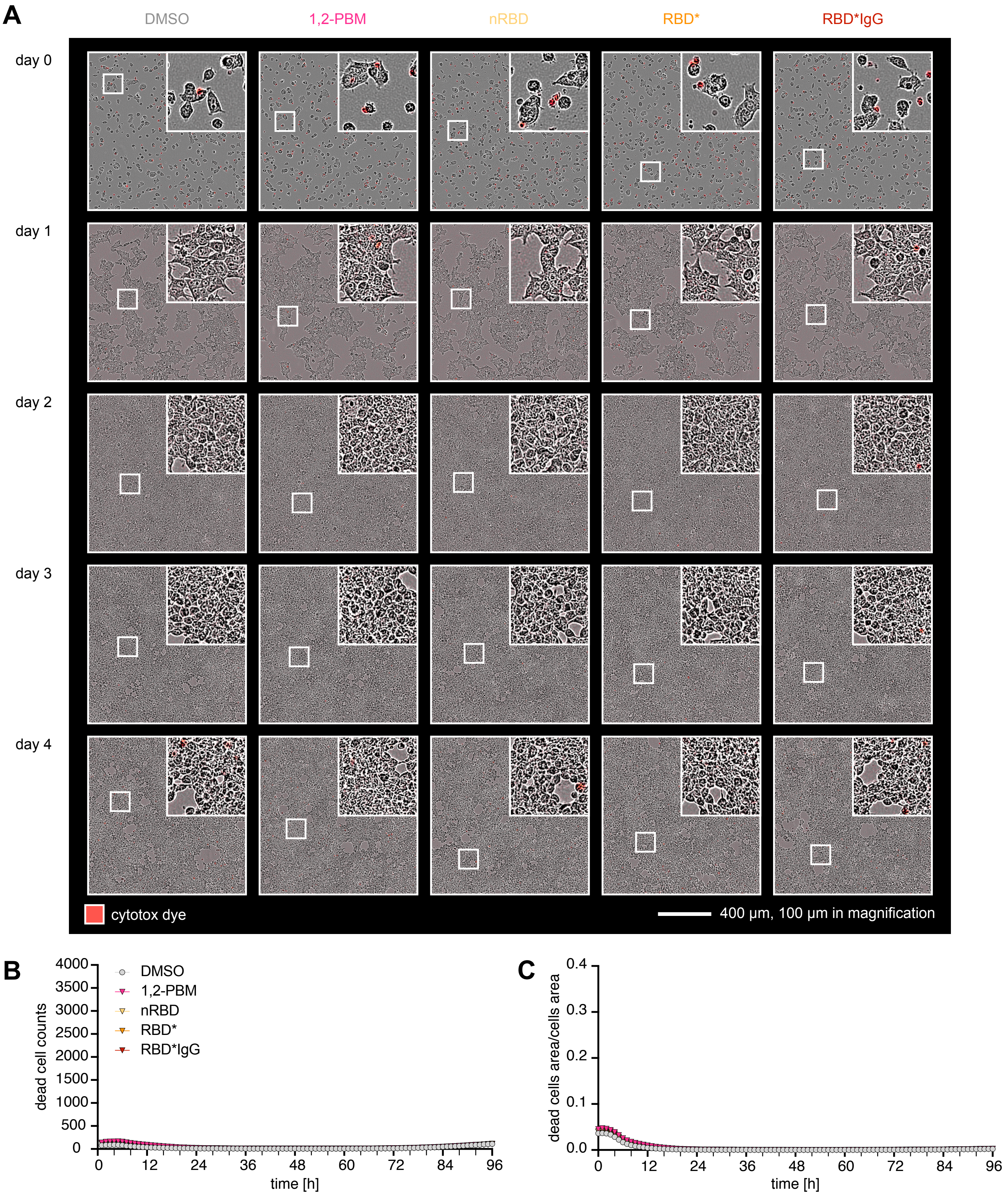

Supplementary Figure S7 | No cytotoxicity upon treatment with 1,2-PBM-generated RBD complexes

A | Representative immunofluorescence data of HEK293 cells upon treatment with 1,2-PBM, nRBD, RBD* or RBD*IgG at the time points stated in the figure. DMSO served as solvent control. The scale bar represents 400 µm in the overview pictures und 100 µm in the magnification shown in the upper right corner of each picture. The smaller square highlights the position from which the magnification is shown. Red signals indicate dead cells. Pictures are representative of 6 individual experiments.

B | Quantification of dead cells in the pictures from **A**. n = 6 for each condition.

C | Quantification of dead cells area per total cells area in the pictures from **A**.

Figure S7 | No cytotoxicity upon treatment with 1,2-PBM-generated RBD complexes

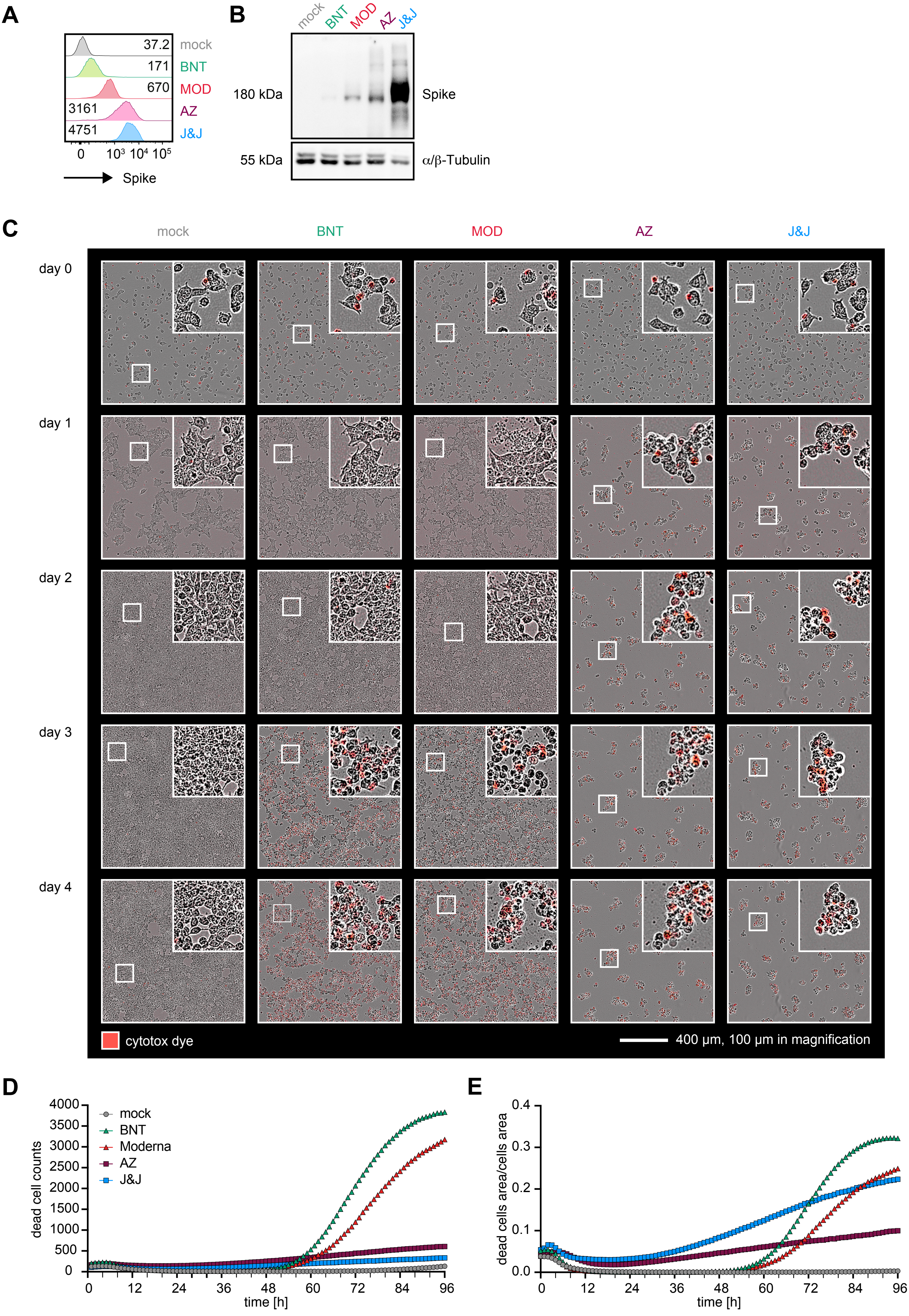

Figure S8 | Comparison of vaccines

Supplementary Figure S8 | Comparison of vaccines

A | Representative SARS-CoV-2 spike protein expression measured by flow cytometry in HEK293 cells upon treatment with either the mRNA vaccines Comirnaty® from BioNtech/Pfizer (BNT) and Spikevax® from Moderna (MOD) or the with the adenoviral vector vaccines Vaxzevria® from AstraZeneca (AZ) or Janssen® from Johnson & Johnson (J&J), respectively. Spike expression was detected by flow cytometry on day 1 after treatment with the respective vaccines.

B | Representative expression of spike protein in HEK293 cells upon treatment with mRNA & vector vaccines as described in **A**, detected two days post treatment by immunoblot.

C | Representative immunofluorescence data of HEK293 cells upon treatment with the indicated mRNA & vector vaccines at the indicated time points. The scale bar represents 400 µm in the overview pictures und 100 µm in the magnification shown in the upper right corner of each picture. The smaller square highlights the position from which the magnification is shown. Red signals indicate dead cells. Pictures are representative of at least 4 individual experiments.

D | Quantification of dead cells in the pictures from **C**. mock n = 6; BNT n = 16; MOD n = 4; AZ n =15; J&J n = 6.

E | Quantification of dead cells area per total cells area in the pictures from **C**.

Figure S8 | Comparison of vaccines

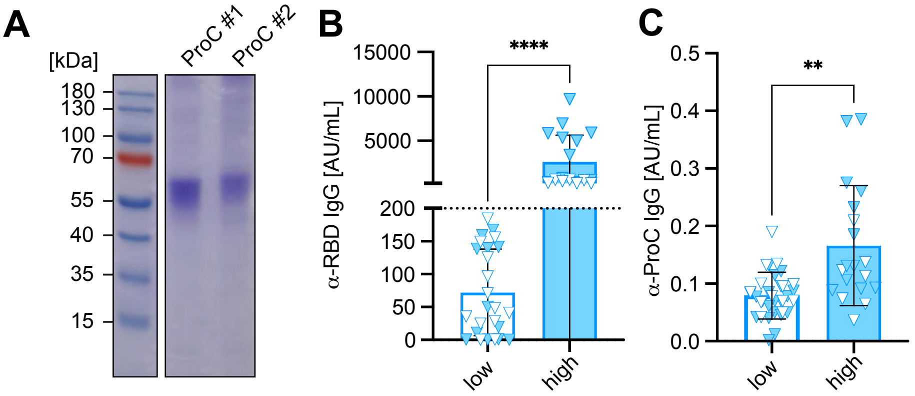

Supplementary Figure S9 | Autoreactive antibodies upon immunization A | Coomassie-stained 10% SDS-page shows two individual preparations of human Protein C (ProC, isoform 10), separated under non-reducing conditions.

B | Human plasma samples from convalescent individuals were divided in two groups with anti-RBD-IgG concentrations >200 AU/mL (high) or <200 AU/mL (low). Samples derived from patients with a mild course of disease are illustrated with white triangles, blue triangles indicate samples from patients with a severe course of disease (intensive care patients). Mean ± SD, statistical significance was calculated by applying the Mann-Whitney-U test.

C | Anti-ProC IgG concentrations in serum samples from **B**. Mean ± SD, statistical significance was calculated by applying the Mann-Whitney-U test.

Figure S9 | Autoreactive antibodies upon immunization
